# Mutualism on the deep-sea floor: a novel shell-forming sea anemone in symbiosis with a hermit crab

**DOI:** 10.1098/rsos.250789

**Published:** 2025-10-22

**Authors:** Akihiro Yoshikawa, Takato Izumi, Takayuki Kanki, Takeya Moritaki, Madoka Kitajima, Naoya Ohtsuchi, Taeko Kimura, Yuxiao Gou, Ryuji Hattori, Mahiro Yumiba, Kotaro Shirai, Michela L. Mitchell, Toshihiko Fujita, Kensuke Yanagi

**Affiliations:** ^1^ Aitsu Marine Station, Center for Water Cycle, Marine Environment and Disaster Management Marine Science Laboratory, Kumamoto University, Kumamoto, Kumamoto, Japan; ^2^ Department of Zoology, National Museum of Nature and Science, Tsukuba, Ibaraki Prefecture, Japan; ^3^ International Center for Island Studies. Amami Station, Kagoshima University, Amami, Kagoshima, Japan; ^4^ Otsuchi Coastal Research Center, Atmosphere and Ocean Research Institute, The University of Tokyo, Otsuchi, Iwate, Japan; ^5^ Faculty of Life Science and Biotechnology, Fukuyama University, Fukuyama, Hiroshima Prefecture, Japan; ^6^ Center for Research and Education of Environmental Technology, Faculty of Engineering, Kyushu University, Motooka, Fukuoka Prefecture, Japan; ^7^ Toba Aquarium, Toba, Mie Prefecture, Japan; ^8^ Enoshima Aquarium, Fujisawa, Kanagawa, Japan; ^9^ Graduate School of Bioresources, Mie University, Tsu, Mie Prefecture, Japan; ^10^ Atmosphere and Ocean Research Institute, The University of Tokyo, Kashiwa, Chiba Prefecture, Japan; ^11^ Faculty of Health and Medical Sciences, Adelaide Medical School, The University of Adelaide, Adelaide, South Australia, Australia; ^12^ Department of Toxinology, Women’s and Children’s Health Network, North Adelaide, South Australia, Australia; ^13^ Graduate School of Science, The University of Tokyo, Bunkyo, Tokyo, Japan; ^14^ Natural History Museum and Institute Chiba Coastal Branch, Katsuura, Chiba, Japan

**Keywords:** co-evolution, Japan, micro-computed tomography scanning, scanning electron microscopy, stable isotope, *Paracalliactis tsukisome* sp. nov.

## Abstract

Interspecific species interactions are fundamental evolutionary forces that shape the traits and adaptive strategies of biological communities. However, their diversity and dynamics in deep-sea ecosystems are poorly understood because of their inaccessibility. Here, we report and describe a newly identified species-specific, hermit crab-associated sea anemone named *Paracalliactis tsukisome* sp. nov. The sea anemone secretes and constructs a unique shell-like structure known as a carcinoecium, which expands the host hermit crab’s living space. Stable isotope analyses (*δ*¹³C and *δ*¹⁵N) suggested that *P. tsukisome* sp. nov. consumes nutritional benefits by consuming host faeces and suspended organic particles from the surrounding environment. Three-dimensional computed tomography imaging elucidated a unidirectional attachment pattern, which was consistently positioned near the shell aperture or carcinoecium edge—a likely adaptation linked to feeding behaviour and carcinoecium formation. The host, *Oncopagurus monstrosus* (Alcock, 1894), substantially benefits from this association, attaining larger body sizes than other *Oncopagurus* species, highlighting the functional role of the carcinoecium as an effective shell enhancement in the deep-sea environment. This study provides the first quantitative evidence of mutualism in carcinoecium-forming associations, highlighting a remarkable example of deep-sea symbiosis and hypothesizing how reciprocal benefits are refined over time, fostering the evolution of carcinoecium-forming abilities and species-specific mutualistic relationships.

## 1. Introduction

Remarkable morphological, behavioural and adaptive strategies are often associated with interspecific biotic interactions such as symbiosis across a wide range of plant and animal taxa [1–3]. Consequently, the trade-offs inherent in these relationships, which drive the evolution of unique traits, have long attracted the attention of evolutionary biologists [4–6].

In the deep sea, carcinoecium-forming (CF) sea anemones of the genera *Calliactis* Verrill, 1869, *Paracalliactis* Carlgren, 1928, and *Stylobates* Dall, 1903 have been recognized for their remarkable interspecific relationship with hermit crabs [7–11]. CF sea anemones settle on gastropod shells inhabited by hermit crabs—often in species-specific relationships—and produce a shell-like structure (carcinoecium) that covers the host shell, thereby expanding the living space of their hosts [10,12–16].

Due to the forward elongation with the growth rate gradient as a vector field along the aperture at a constant angle and shape, which is essential for the formation of a spiral gastropod shell [17], the CF ability is especially remarkable in cnidarians, which are the most closely related animal group of bilateral animals [18]. Although the bilateral symmetry of Anthozoa has been observed in several morphologies (e.g. number of siphonoglyphs and tentacles) and developmental patterns (e.g. arrangement of mesenteries) [19,20], unidirectional movement in bilateral or cephalized animals has not been previously documented in cnidarians. Therefore, the CF ability of the sea anemone is valuable for further understanding the evolutionary origin and mechanisms of the unidirectional movement in bilateral or cephaline characteristics of bilateral animals.

However, the key evolutional forces driving CF ability and species-specific symbiosis on the deep-sea floor remain unclear because of the inaccessibility of deep-sea habitats and taxonomic lag in describing deep-sea sea anemone fauna [12,13,21,22]. In this study, therefore, to understand the key forces influencing the evolution of CF ability in sea anemones, we investigated the symbiotic merits of the newly identified sea anemone *Paracalliactis tsukisome* sp. nov. (Cnidaria: Actiniaria: Hormathiidae) and its host hermit crab *Oncopagurus monstrosus* (Alcock, 1894).

This novel CF sea anemone, *Paracalliactis tsukisome* sp. nov., is the ninth valid species in the *Paracalliactis* genus found on the shells inhabited by *O. monstrosus* at depths of 192−470 m around the Pacific coast of Japan. The genus *Paracalliactis* previously comprised eight valid species, one questionable species, *Paracalliactis involvens* (McMurrich, 1893), and one undescribed species, *Paracalliactis* sp. (ms. n, described by Gusmão [9] as *P. niwa*), all found exclusively on shells inhabited by hermit crabs. Most *Paracalliactis* species inhabit the deep-sea floor (approx. 200−4700 m depth), while two species, *P. rosea* (Hand, 1975) and *P. sinica* (Pei, 1982), have been recorded in shallow to deep depths of 50−3000 m [9,23,24] and at shallow depths of 39−40 m, respectively. This novel CF sea anemone was provisionally found only in association with *O. monstrosus*. Other CF sea anemones often establish a species-specific association [13,14,16,22]; the relationship between *Paracalliactis tsukisome* sp. nov. and *O. monstrosus* appears to be a species-specific association. Therefore, it may prove to be a suitable model to understand the evolution of the unique traits described above, as well as the evolution of obligate symbiosis in the deep-sea benthic community.

As a symbiotic merit of the sea anemone, an increase in the food supply from the environment or food residuals of hermit crabs is a well-known representative hypothesis regarding their advantages [25]. Previous studies have discussed this possibility based on indirect data, such as the contents of the sea anemone’s gut and the host hermit crab’s mobility [25–27], but have not tested directly. Therefore, by performing carbon (*δ*^13^C) and nitrogen (*δ*^15^N) stable isotopic analyses of *P. tsukisome* sp. nov. and its host hermit crab *O. monstrosus*, the feeding habits of *P. tsukisome* sp. nov. were documented to understand the symbiotic advantages in terms of its feeding potential. Considering the ecological significance of the attachment position in feeding ecology, which was discussed in a previous study [13], a three-dimensional analysis was also performed. The relationship between the attachment position and carcinoecium formation, which may lead to the elongation of the shell aperture, is also discussed.

The benefits for host hermit crabs in the CF association are also largely unknown. Given that their growth and reproductive success mainly depend on shell availability and quality [28–30], the availability and quality of the carcinoecium are likely to be directly related to these benefits. However, shell change behaviour in the hermit crab *Pagurodofleinia doederleini* Doflein, 1902 has been observed, even in association with the CF sea anemone *Stylobates calcifer* Yoshikawa & Izumi, 2022 [13]. Thus, it is unclear whether the carcinoecium acts as a ‘shell substitution’ for the host hermit crab, and this speculation remains controversial. In this study, we hypothesized that if the carcinoecium produced by *P. tsukisome* sp. nov. functions as a portable shelter along with the real gastropod snail shells of *O. monstrosus*, this hermit crab species could grow bigger than other opportunistic-symbiotic and non-symbiotic *Oncopagurus* Lemaitre, 1996 species in the deep sea. This hypothesis was tested to determine the benefit of carcinoecium hosting hermit crabs by comparing the carapace-shield length of *O. monstrosus* and 23 other *Oncopagurus* species.

Cumulatively, this study provides evidence of mutualism in iconic relationships in the sea and is the first to demonstrate symbiotic benefits in a CF relationship, along with quantifiable data. Furthermore, it contributes to our understanding of the interspecific interactions that drive unique co-evolutionary patterns within deep-sea communities.

## 2. Material and methods

### 2.1. Sample collection, preservation and behavioural observation

**Figure 1.**
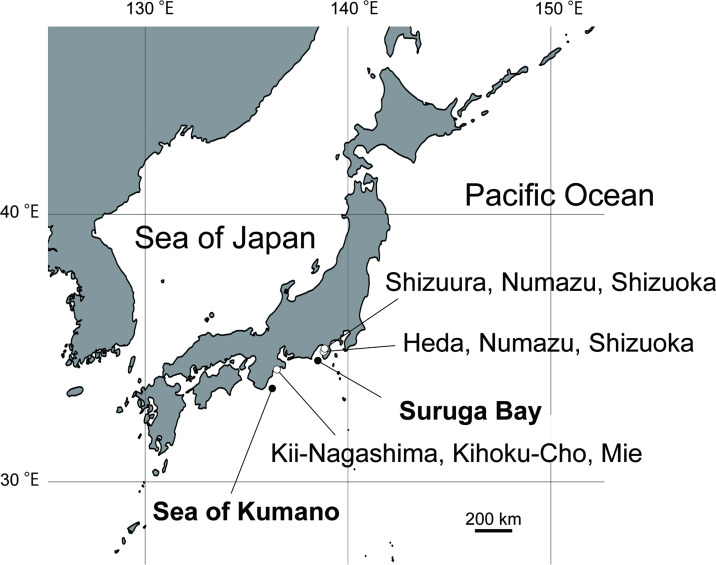
*Paracalliactis tsukisome* sp. nov. sampling locations. The trawled sea area (solid circles) and location of the fishing ports at Kii-Nagashima, Kihoku-Cho, Mie and at Heda and Shizuura, Numazu, Shizuoka where *P. tsukisome* sp. nov. was caught (open circles).

The newly identified sea anemone was found on the snail shells of the hermit crab *O. monstrosus*. A total of 36 specimens were collected between November 2017 and March 2024 through beam trawling operations conducted in several localities: the Sea of Kumano at depths of 192−204 m; Kii-Nagashima in Kihoku-cho, Mie, Japan; Suruga Bay along the Pacific coast of Honshū at approximately 320−470 m depth; and Heda and Shizuura in Numazu, Shizuoka, Japan, 300−350 m (figure 1; electronic supplementary material, table S1). Detailed information on the collection locations, dates, depths and specimen usage is provided in electronic supplementary material, table S1.

The sea anemones were anaesthetized using isotonic magnesium chloride (MgCl₂) prior to morphological and histological observations, DNA sampling and preservation. Specimens were fixed in 10%−20% formalin prepared in seawater, rinsed in running freshwater overnight (approximately 12 h), and preserved in 70% ethanol. In several specimens, the body column was excised from the snail shell and dissected to obtain the mesenteries, sphincter muscles and tentacles for morphological examination. The holotype (NSMT-Co 1822) and nine paratypes (NSMT-Co 1823, CMNH-ZG 10596-10600, CMNH-ZG 10619, CMNH-ZG 10621 and CMNH-ZG 10622), together with their host hermit crabs and inhabited snail shells, were deposited at the National Museum of Nature and Science, Tsukuba (NSMT), Ibaraki, and the Coastal Branch of the Natural History Museum and Institute, Chiba (CMNH), respectively. The procedure for specimen preparation followed the previous taxonomic study on CF sea anemones in interspecific relationships with deep-sea hermit crabs [13].

To record the activities of the newly identified deep-sea species, a living hermit crab and sea anemone pair were transported to the laboratory at Otsuchi Coastal Research Centre, Atmosphere and Ocean Research Institute (AORI), The University of Tokyo, where they were housed in a marine aquarium kept at 10°C, fed krill (*Euphausia* spp.), and video recorded. Subsequently, the coloration and the expansion–contraction behaviours of an individual (CMNH-ZG 10618) were observed using a Canon EOS Kiss X9i from 28 to 31 June 2022.

### 2.2. Preparation of histological sections

To examine the internal morphology, the upper portion of the sea anemone column was removed from the shells inhabited by the holotype (NSMT-Co 1822) and the paratype (CMNH-ZG 10597). From this tissue, mesenteries, sphincter muscles and tentacles were dissected. Samples were subjected to a dehydration series using ethanol at concentrations of 80% and 90% for 2 h each and 99% for 1 h, followed by two treatments in absolute ethanol for 30 min. The tissues were then cleared in a xylene sequence (ethanol-xylene, xylene I and xylene II; 20 min each), embedded in paraffin (xylene-paraffin for 30 min, paraffin I for 2 h and paraffin II for more than 2 h), microtomed into 8 μm thick slices, mounted on slides and stained using Masson’s trichrome technique [31].

### 2.3. Cnidae observation

For cnidae analysis, tissue fragments (tentacle, actinopharynx, mesenterial filament, column, limbus and acontia) from the holotype (NSMT-Co 1822) and paratype (CMNH-ZG 10596) were each mounted in a 50% glycerine–seawater solution on microscope slides. Observations were made using differential interference contrast (DIC) microscopy [32]. Cnidae types were identified based on the classification system proposed by Mariscal [33] and were measured using ImageJ software, version 1.49 [34].

### 2.4. Scanning electron microscopy of the carcinoecium

The surface structure of the carcinoecium (paratype NSMT-Co 1823) was observed using a low-voltage scanning electron microscopy (Lv-SEM; TM4000Plus, Hitachi High-Technologies, Tokyo, Japan). Because the carcinoecium is secreted directly under the pedal disc by the sea anemone and is not easily removed, we could only observe its inner surface.

### 2.5. Molecular phylogenetic analysis

Total DNA was isolated from the tissue samples of three specimens (holotype NSMT-Co 1822, paratype NSMT-Co 1823 and CMNH-ZG 10596) using a High Pure PCR Template Kit (Roche, Darmstadt, Germany). Fragments of approximately 700 bp (COXIII), 500 bp (mitochondrial 16S rRNA), 800 bp (12S rRNA), 1700 bp (nuclear 18S rRNA) and 3200 bp (28S rRNA) were amplified via polymerase chain reaction (PCR). Each reaction (25 μl total) contained 2.5 μl of each primer (forward and reverse), 2.5 μl of Ex TaqTM buffer, 2.0 μl dNTP mix, 0.13 μl of Ex Taq DNA polymerase (TaKaRa, Japan) and 14.87 μl of sterile distilled water. The thermal cycling protocol involved initial denaturation at 94°C for 3 min, followed by 35 cycles of 94°C for 45 s, annealing at various temperatures for 90 s, and 72°C for 120 s, concluding with a final extension at 72°C for 10 min. The amplified products were visualized on a 1% agarose gel, purified with the High Pure PCR Product Purification Kit (Roche), and sequenced by Eurofins Genomics Inc. using either only PCR primers (COXIII, 12S, 16S) or specific sequencing primers in addition to PCR pairs (18S, 28S). Sequence assembly was performed using GeneStudio v. 2.2.0.0, and data were submitted to the DNA Data Bank of Japan (electronic supplementary material, table S2).

To determine the phylogenetic position of the newly identified sea anemone, phylogenetic reconstructions were conducted within the superfamily Metridioidea. The sequences of Metridioidea, except for *P. tsukisome* sp. nov. analysed in the present study, and Actinioidea which acted as an outgroup for the phylogenetic analysis of the suborder level, were obtained from GenBank (electronic supplementary material, table S2). The dataset was aligned using MAFFT v. 7.402 [35] under the default settings. Ambiguously aligned regions were eliminated using Gblocks v. 0.91b [36].

The aligned dataset was processed in Kakusan4 [37] to assess its substitution models for downstream analyses using RAxML and MrBayes. Maximum likelihood (ML) analysis was performed with RAxML-VI-HPC [38] using the GTR+Γ model as suggested by Kakusan4, and node support was estimated via 1000 bootstrap replicates. Bayesian inference (BI) was executed in MrBayes v. 3.2.6 [39] with the following substitution models: GTR+Γ for COXIII, 12S, and 28S; HKY85+Γ for 16S; and SYM+Γ for 18S. Two independent Markov chain Monte Carlo (MCMC) runs were performed for 5 000 000 generations, with sampling every 100 generations. The first 25% of samples were discarded as burn-in, and convergence was monitored by assessing the average standard deviation of the split frequencies (ASDSFs) every 100 000 generations. The final consensus trees were visualized using FigTree v. 1.4.4 [40], with nodes having bootstrap support below 50% or posterior probabilities under 0.50 removed manually.

Genetic divergence at both the interspecific and intraspecific levels was calculated using the Kimura two-parameter (K2P) model [41] implemented in MEGA X [42]. Interspecific distances were evaluated among closest relatives or congeners in the phylogenetic tree. The procedure for the molecular phylogenetic analysis generally followed that of the previous taxonomic study on CF sea anemones [13].

### 2.6. Carbon and nitrogen isotope analyses

The stable *δ*^13^C and *δ*^15^N isotope values for the 15 pairs of *P. tsukisome* sp. nov. and *O. monstrosus* were examined to determine the feeding interactions of the associated animals. The muscle tissues, which were fixed in 10%−20% formalin: seawater and preserved in 70% ethanol, were analysed. The tentacle muscles, including the tentacular circular and longitudinal muscles and sphincter muscle of *P. tsukisome* sp. nov. and the abdomen muscle of *O. monstrosus*, served as species samples.

The muscle tissue samples were dried at 50°C for 24 h to remove water. Lipids were removed using a chloroform/methanol (2 : 1) solution and tissue ground to a fine powder [43]. Approximately 2.0 ± 0.1 mg of muscle powder was sealed in a tin capsule. *δ*^13^C and *δ*^15^N analyses were performed according to a previously reported protocol [44], using an isotope ratio mass spectrometer (IsoPrime100, IsoPrime) interfaced with an elemental analyser (Vario Micro Cube, Elementar) installed at the Atmosphere and Ocean Research Institute, University of Tokyo. The *δ*^13^C and *δ*^15^N were expressed in *δ* notation and are reported relative to Vienna Pee Dee Belemnite and N_2_ in air for *δ*^13^C and *δ*^15^N respectively, defined using equation (2.1) as follows:


(2.1) $$\delta^{13}C\;\text{or}\;\delta^{15}N\;=\;(\frac{R_{\text{sample}}}{R_{\text{standard}}}-1)\;\times 1000\;(\text{‰}),$$

where *R* is ^13^C/^12^C or ^15^N/^14^N. The isotopic composition was calibrated against a commercial standard (L-Alanine AZ101-SS13, *δ*^13^C = −19.6‰ and *δ*^15^*n* = 13.7‰, Shoko Science), producing a reproducibility greater than 0.20‰ for *δ*^15^N and 0.28‰ for *δ*^13^C. The above method of carbon and nitrogen isotope analysis was conducted by following the method described by Zhao *et al*. [44].

Moreover, to discuss the trophic interaction of sea anemones and their host hermit crabs, the *δ*^13^C and *δ*^15^N values of the sediment and suspended particles from the same region, Kumano Nada, reported by Nishimoto *et al*. [45], were used as reference data for the sampling region of the present study. The *δ*^13^C and *δ*^15^N values of the suspended particles of this region were estimated to be 22 and +3 to +5‰, respectively, based on the suspended feeding species *Nypamodiolus japonicus* (T. Habe, 1976) reported by Nishimoto *et al*. [45]. The estimated values of the faeces and exuviae of hermit crabs were calculated using a trophic fractionation factor of +1.68‰ in *δ*^13^C and +3.4‰ in *δ*^15^N from estimated hermit crab’s food and +1.2‰ in *δ*^13^C and −5.88‰ in *δ*^15^N from hermit crab’s muscle for following the value on the invertebrate animals reported by Reid *et al*. [46].

### 2.7. Three-dimensional analysis of the attachment direction and position

The InspeXio SMX-225CT FPD HR (Shimadzu Co., Ltd., Kyoto, Japan) at the National Museum of Nature and Science, Tsukuba, Ibaraki, Japan, was used to obtain thethree-dimensional data of *P. tsukisome* sp. nov. First, the examined samples were post-fixed in 0.05 mol l^−1^ (N/10) aqueous potassium iodide solution for 1.5 to 12 h at room temperature (approximately 25°C) without removing the sea anemone from the host shells. Then, micro-computed tomographic (micro-CT) scanning was conducted with the following parameters: source voltage = 115 kV, source current = 70 μA, exposure time for one frame = 0.79 s, total number of frames = 760, total time for scanning = 10 min, and detector size = 1024 × 1024 pixels. All section images were evaluated and reconstructed into a three-dimensional structure using the commercial software package VGSTUDIO MAX 3.3.6 (Volume Graphics, Heidelberg, Germany). The siphonoglyphs aligned with the directive axis were detected using the 0.5 mm tips of the region of interest (ROI) detection system using the same software. The reconstructed three-dimensional images and ROI images of the siphonoglyphs were separately exported as a PLY file.

The following landmarks were set on the reconstructed three-dimensional images to measure the attachment position of the sea anemone: A, midpoint between two upper tips of the detected siphonoglyph; B, closest point on the surface of the host snail shell from point A; C, upper tip of the outer lip of the host shell or carcinoecium edge; D, lower tip of the outer lip of the host shell or carcinoecium edge; E, intersection of the approximation plane of the siphonoglyph and the straight line CD; F, intersection of the straight line AB and the approximation plane of the shell aperture or carcinoecium edge (G).

Angles 1 and 2 were measured. Angle 1 is the ∠CBE; when point F is on the CD side, the value of Angle 1 is positive. In this case, it can be inferred that the sea anemones’ directive axis is towards the shell aperture. When point E is located on the CD side, the value of Angle 1 is negative (electronic supplementary material, figure S1, I). In this case, the directive axis of the sea anemone is directed towards the shell apex. Additionally, the closer the absolute value of Angle 1 is to 0°, the more the directive axis faces the upper tip of the shell aperture (point C).

Angle 2 is the angle formed between the directive axis of the sea anemone and its intersection with the shell aperture of the host snail shell, i.e. ∠AFG (electronic supplementary material, figure S1, II). When the value of Angle 2 is less than 90°, it was inferred that the sea anemone was attached to the shell face while oriented upward (dorsal side of the host hermit crab). When the value of Angle 2 is greater than 90°, the sea anemone is oriented towards the substrate (ventral side of the host hermit crab).

All landmarks were set using the open-source software MeshLab 2016.12 [47], and angles were calculated using the Julia 1.5.3 program [48]. The examined components are schematized in electronic supplementary material, figure S1.

### 2.8. Size comparison of *Oncopagurus monstrosus* and other *Oncopagurus* species

In total, the body sizes of 1107 specimens of 24 *Oncopagurus* species, including *O. monstrosus*, were compared to test the following hypothesis: ‘*if the carcinoecium produced by* P. tsukisome *sp. nov. functions as a portable shelter along with the real gastropod snail shells of* O. monstrosus, *this hermit crab species could grow bigger than other non-symbiotic* Oncopagurus *species in the deep sea*’. The carapace shield length (SL) of the hermit crabs, an index of body size, was measured using a calliper with a precision of 0.01 mm or sourced from a previous study by Lemaitre [49].

In total, 194 *O. monstrosus* individuals (43 non-ovigerous females, 17 ovigerous females and 134 males) were examined in this study. Newly collected specimens collected during this study include 32 individuals (1 non-ovigerous female and 31 males) deposited in the National Museum of Nature and Science (Register nos.: NSMT-Co 1822, NSMT-Co 1823) and the Museum and Institute of Chiba (CBM ZC 2891-2914, CBM ZC 2916, CBM ZC 2918-2923) (electronic supplementary material, table S1). In addition, 33 specimens (7 non-ovigerous females, 3 ovigerous females and 23 males) previously deposited in these museums were also measured: NSMT-Cr 33027-33032 at the National Museum of Nature and Science, and CBM ZC 352, CBM ZC 3236, CBM ZC 4534, CBM ZC 6285, CBM ZC 10166, CBM ZC 10561, and KH05-01 Actinaria-22, 23 at the Museum and Institute of Chiba. Data for the remaining 129 individuals were sourced from a previous study by Lemaitre [49]. Moreover, *O. monstrosus* was categorized as possibly a specific association with actinian species by Lemaitre as ‘*Gastropod shells usually with actinian attached to shell*’ [49].

Ten specimens of *Oncopagurus cidaris* Lemaitre, 1996 (four ovigerous females and six males) (Register nos.: QM W16496, QM W16506, QM W16508, QM W16596, QM W20644, QM W20645) and 20 specimens of *Oncopagurus indicus* (Alcock, 1905) (1 non-ovigerous female, 1 ovigerous female and 18 males) deposited in the Queensland Museum were also measured in this study (Register nos.: QM W16590, QM W16599, QM W16600). All other data related to the length of *Oncopagurus* species were obtained from published literature [49–52]. The museum specimens of *Oncopagurus* species examined in the present study are listed in electronic supplementary material, table S3.

Moreover, the calcification of the symbiotic style was based on Lemaitre’s descriptions [49] and observations during this study. Seven species were categorized as opportunistic species associated with Anthozoan polyps or *Hydractinia* colonies—*Oncopagurus africanus* (de Saint Laurent, 1972), *Oncopagurus elongatus* Lemaitre, 2014, *Oncopagurus gracilis* (Henderson, 1888), *Oncopagurus haigae* (de Saint Laurent, 1972), *O. indicus*, *Oncopagurus orientalis* (de Saint Laurent, 1972), and *Oncopagurus stockmani* Zhadan, 1997. *Oncopagurus bicristatus* (A. Milne-Edwards, 1880) was categorized as CF and/or opportunistic Anthozoan-associated species because although an association of this species with *Paracalliactis valdiviae* has been reported, this association did not indicate species-specific symbiosis [49,53]. *Oncopagurus tuamotu* (Lemaitre, 1994) was categorized as possibly a specific association with Anthozoan polyps based on its description by Lemaitre as ‘*Gastropod shells, usually with Anthozoan polyps growing on the shell*’ [49]. The remaining 13 species were categorized as non-symbiotic species.

For statistical analysis, Welch’s *t*-test with Bonferroni adjustments was performed to compare the body sizes of each sex of *O. monstrosus* with those of other *Oncopagurus* species. We analysed species with more than two individuals. Therefore, we compared 14, 13 and 20 *O. monstrosus* specimens of non-ovigerous females, ovigerous females and males, respectively. The following Bonferroni adjustments were considered in each analysis: non-ovigerous female, *p* = 0.05/14 = 0.004; ovigerous female, *p* = 0.05/13 = 0.004; all females, *p* = 0.05/15 = 0.003; male, *p* = 0.05/20 = 0.003. Welch’s *t*‐test was conducted to compare the body size of the examined specimens of *O. monstrosus* that are confirmed association with *P. tsukisome* sp. nov. with those reported in a previous study by Lemaitre [49], which were probably related to Actiniarian species. All statistical analyses were performed with R v. 3.4.0 [54].

## 3. Results

### 3.1. Taxonomic description

Order ACTINIARIA Hertwig, 1882
Suborder ENTHEMONAE Rodriguez & Daly, 2014
Superfamily METRIDIOIDEA Carlgren, 1893
Family HORMATHIIDAE Carlgren, 1932
Genus *Paracalliactis* Carlgren, 1928
(New Japanese name: Tsukisome-isoginchaku-zoku)

*Genus diagnosis* (modified from Gusmão *et al*. [55]; additions in bold): Hormathiidae with well-developed, broad and asymmetric pedal disc but not bilobed as in some *Calliactis* species. Pedal disc slightly wider than oral disc; secretes carcinoecium that may or may not project beyond shell aperture. Column divisible into scapus and scapulus; smooth or with distal tubercles that form a complete or **weak corona**; may have a thin, easily deciduous cuticle. No cinclides. Column with basitrichs and microbasic *p*-mastigophores B1. Tentacles hexamerously arranged, approximately the same number as mesenteries proximally, their longitudinal muscles ectodermal. Strong, mesogleal marginal sphincter muscle present. About the same number of mesenteries proximally and distally; six pairs of perfect and sterile mesenteries, including two pairs of directives each associated to a siphonoglyph. Retractor and parietobasilar muscles are weak. Species live attached to gastropod shells inhabited by hermit crabs with oral disc positioned directed away from the aperture of the shell (dorsally) or below the aperture of the shell (ventrally).

*Type species: Paracalliactis valdiviae* Carlgren, 1928.

*Valid species: Paracalliactis azorica* Doumenc, 1975; *P. consors* Verrill, 1882; *P. involvens* McMurrich, 1893; *P. michaelsarsi* Carlgren, 1928; *P. obvolva* [8]; *P. rosea* Hand, 1975; *P. sinica* Pei, 1982; *P. stephensoni* Carlgren, 1928; *P. valdiviae* Carlgren, 1928; *P. tsukisome* sp. nov.

**Figure 2.**
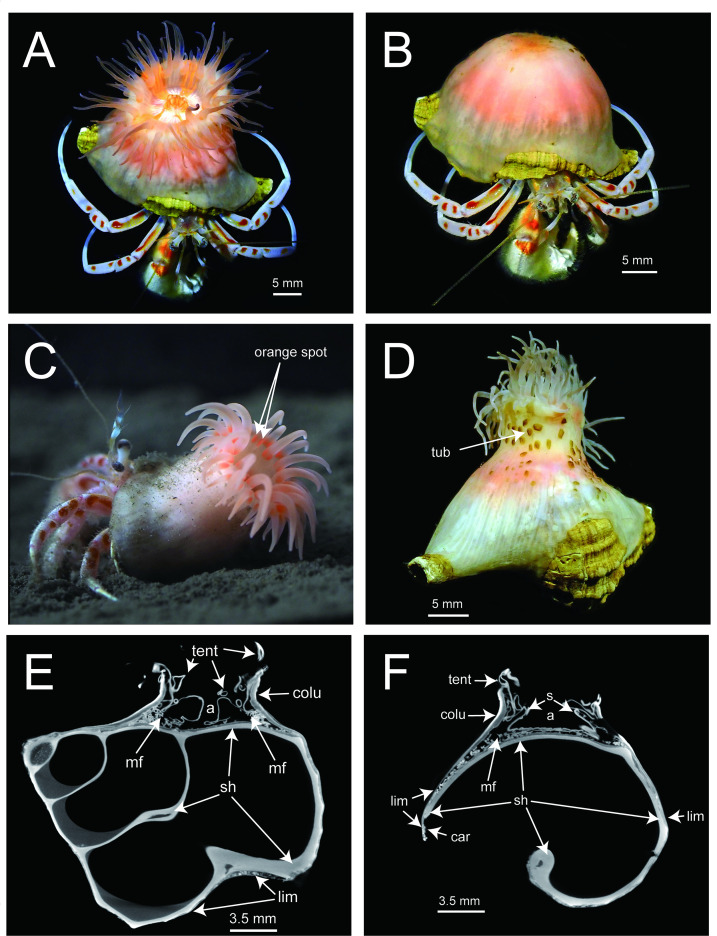
External and internal morphology of *Paracalliactis tsukisome* sp. nov. Upper views of the living specimen (holotype: NSMT-Co 1822) with open (A) and shrunken (B) tentacles. (C) Live specimen used for behavioural video recording (CMNH-ZG 10618). (D) Posterior view of the fixed holotype (NSMT-Co 1822). (E,F) Micro-CT-scanned views of the specimen (CMNH-ZG 10621) from the shell tip–umbilicus plane of the host snail shell (E) and the vertical plane (F). Abbreviations: a, actinopharynx; mf, mesenterial filament; tent, tentacle; colu, column; lim, limbus; os, original shell; car, carcinoecium; tub, tubercle; s, siphonoglyph.

**Figure 3.**
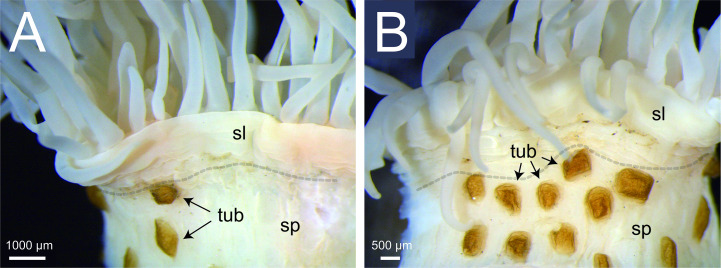
Enlarged posterior view of the column of *Paracalliactis tsukisome* sp. nov. (holotype, NSMT-Co 1822) indicating divisible scapus and scapulus with irregularly numbered tubercles forming a weak corona (scapus and scapulus division represented by a transparent grey dotted line). (A) The divisible scapus and scapulus on the column and scapus are smooth until the distal part (the view from the other side of the shell aperture). (B) Irregularly numbered tubercles on the scapulus covered by a thin, brown cuticle, form a weak corona (the view from the umbilicus side of the shell). Abbreviations: sp, scapus; sl, scapulus; tub, tubercle.

**Figure 4.**
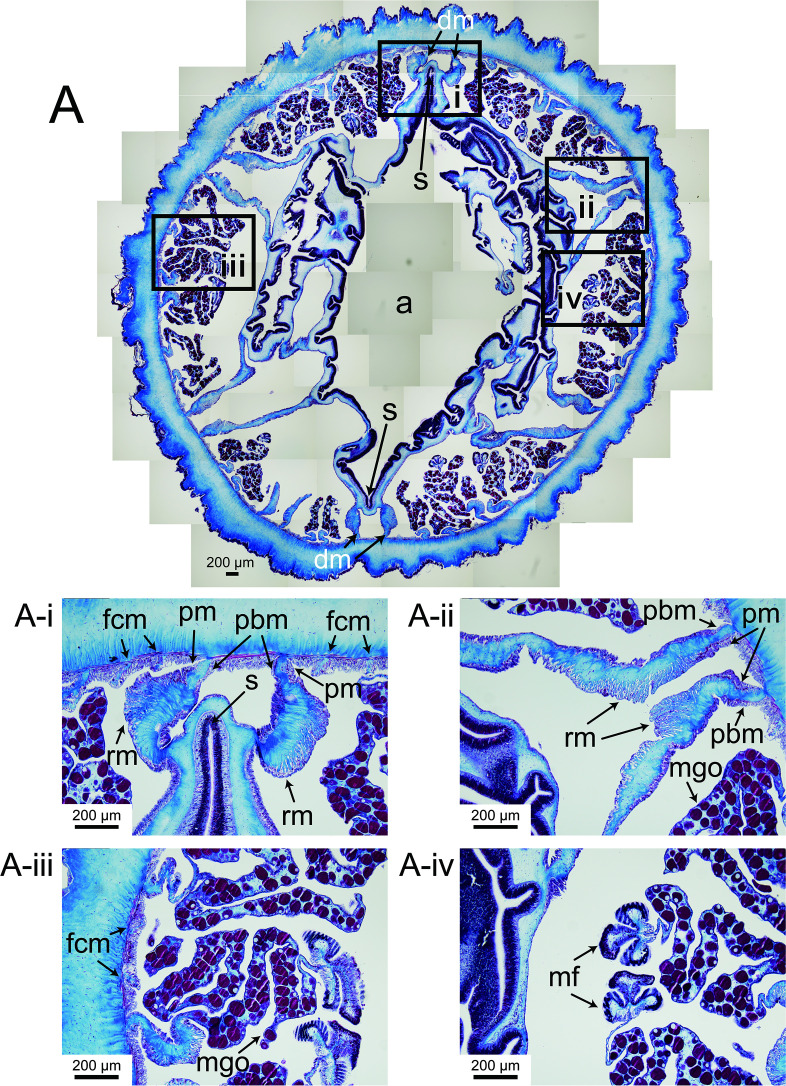
Histological section of a column of *Paracalliactis tsukisome* sp. nov. (holotype: NSMT-Co 1822). (A) Entire transverse serial section of the actinopharynx. (A-i) Enlarged view of the section through the actinopharynx showing the directives (first mesenteries) and siphonoglyph. (A-ii) Enlarged view of the first mesentery and its retractor muscle. (A-iii) Enlarged view of the matured ovarian cyst of the second and third mesenteries. (A-iv) Enlarged view of the mesenterial filament of the second and third mesenteries. Abbreviations: a, actinopharynx; fcm, a fourth cycle mesentery pair; mf, mesenterial filament; mgo, matured gamete vesicle and oocytes (eggs); pm, parietal muscles; pbm, pariental basilar muscle; rm, retractor muscle; s, siphonoglyph.

**Figure 5.**
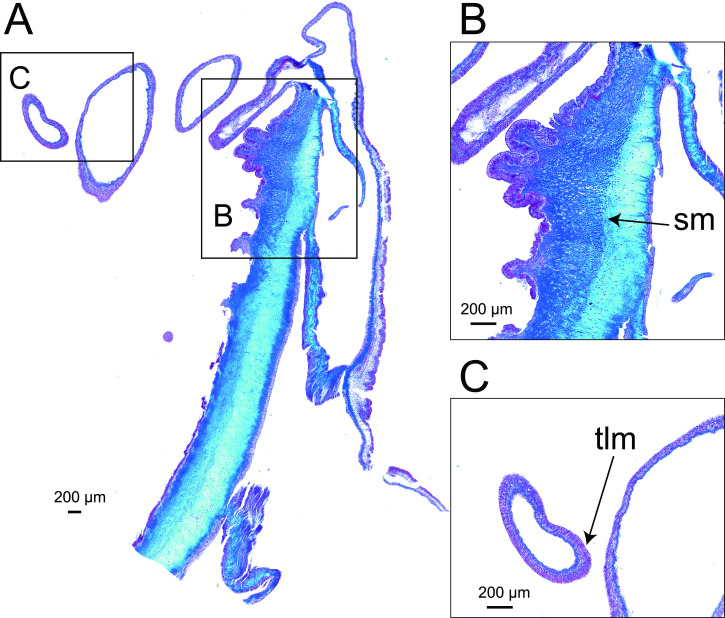
(A) Internal anatomy of *Paracalliactis tsukisome* sp. nov. (holotype: NSMT-Co 1822). (B) Longitudinal section of the diffuse and strong, mesogloeal marginal sphincter muscle. (C) Transverse section of a tentacle (arrow). Abbreviations: sm, sphincter muscle; tlm, tentacular longitudinal muscle.

**Figure 6.**
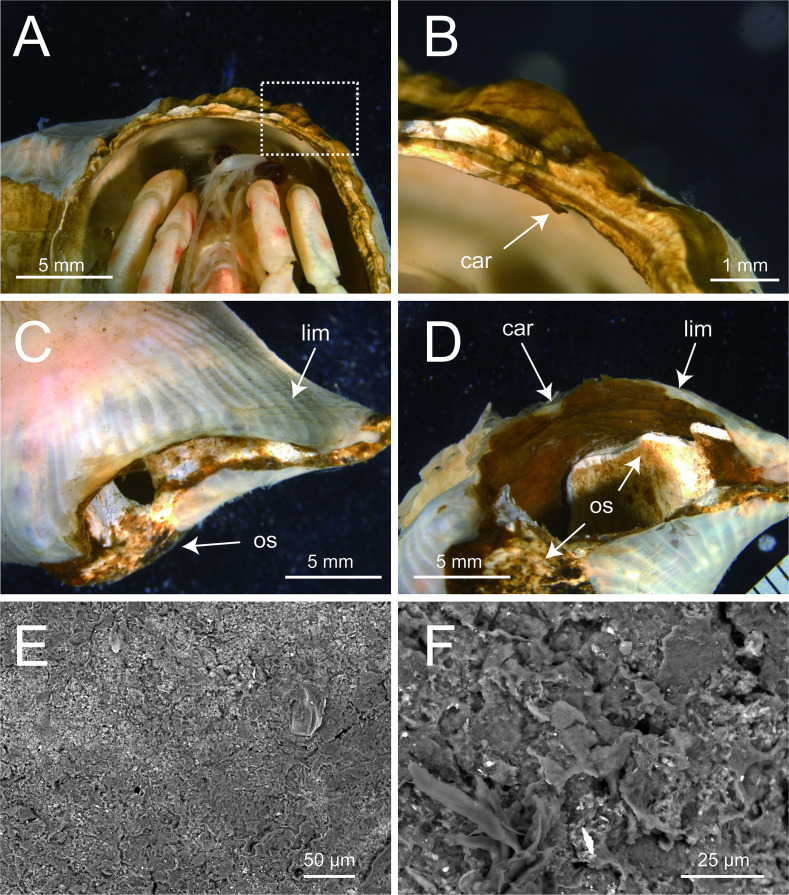
External morphology of the carcinoecium produced by *Paracalliactis tsukisome* sp. nov. (holotype: NSMT-Co 1822). (A,B) Dorsal/ventral view of the carcinoecium on the shell. (C,D) Dorsal/ventral view of the carcinoecium on the host snail shell (paratype: NSMT-Co 1823). (E,F) Scanning electron microscope (SEM) images of the inner surface of *P. tsukisome* sp. nov. (paratype: NSMT-Co 1823) at ×300 (E) and ×1000 (F) magnification. Abbreviations: car, carcinoecium; lim, limbus; os, original shell.

*Paracalliactis tsukisome* sp. nov. Yoshikawa, Izumi & Yanagi

(Japanese name: Tsukisome-isoginchaku)

(figures 2–6, electronic supplementary material, figures S2, S3)

*ZooBank ID (LSID*): urn:lsid:zoobank.org:pub:E75A046F-3E5C-45F7-BBFE-439368A41BF8

*Material examined*. **Holotype**: NSMT-Co 1822, collected from the Sea of Kumano, off the coast of the Kii Peninsula, Honshu Japan (34°05.5′ N 136°32.2′ E), using the bottom trawl net with the fishing trawler Jinsho-maru, on 3 June 2020, at a depth of approximately 300 m.

**Paratypes:** NSMT-Co 1823, CMNH-ZG 10596-10601; collected from the Sea of Kumano, off the coast of the Kii Peninsula, Honshu Japan (34°05.5′ N 136°32.2′ E), using the bottom trawl net with the fishing trawler Jinsho-maru, on 3 June 2020, at a depth of approximately 300 m; CMNH-ZG 10619, 10621 and 10622; collected from Suruga Bay, the coast of Heda, Numazu, Shizuoka, Japan, using a trawl net on the fishing trawler Hinode-maru, on 18 February 2018, at a depth of approximately 300 m.

**Other specimens:** CMNH-ZG 10602-10618; collected from the Sea of Kumano, off the coast of the Kii Peninsula, Honshu Japan (34°04.4′ N 136°31.6′ E), using the bottom trawl net with the fishing trawler Jinsho-maru, on 8 June 2020, at a depth of 320−470 m; CMNH-ZG 10620; collected from the Sea of Kumano, off the coast of the Kii Peninsula, Honshu Japan, using a beam trawl on the T/S Seisui-maru, on 9 November 2017, at a depth of 192−204 m. CMNH-ZG 10623-10628; collected from Suruga Bay, the coast of Numazu, Shizuoka, Japan, Japan, using a trawl net on the fishing trawler Hinode-maru, on 10 April 2020, at a depth of approximately 300 m. NSMT-Co 1920, collected from Suruga Bay, the coast of Heda, Numazu, Shizuoka, Japan, using a trawl net on the fishing trawlers, on 5 March 2024, at a depth of 300−350 m. All types and non-types are summarized in electronic supplementary material, table S1.

*External morphology*. Pedal disc: slightly concave conforming to the shape of a snail shell (figure 2A–D), thin, smooth, flat; diameter of the pedal disc depended on the shell’s length, wider than oral disc: edge smooth with an irregular outline dependent on shape of the snail shell (figure 2A,B,D), covering the entire shell except for the part underside of the *O. monstrosus* (figure 2D–F).

Column: cylindrical on the shells, at 8.9 mm in height (fixed NSMT-Co 1822), divisible into scapus and scapulus (figure 3). Scapus smooth until distal part, close to the scapulus, irregularly numbered tubercles form a weak corona (figure 3). Some tubercles covered by a thin, brown cuticle (figures 2D and 3A,B). No cinclides (figure 2A–D).

Oral disc: cylindrical with an oval mouth at 16.1 mm in diameter (live NSMT-Co 1822) with two prominent siphonoglyphs (figure 4A), central mouth weakly sharp in the live and fixed specimens (figure 2A, electronic supplementary material, video S1). Actinopharynx shallowly ribbed with two symmetrical siphonoglyphs (figure 4A).

Marginal tentacles thin and pointed with length 3.1−9.9 mm and diameter 0.66−1.35 mm (fixed NSMT-Co 1822). Tentacles number 90 to 95 (electronic supplementary material, table S1).

*Internal morphology*. Mesenteries hexamerously arranged in four cycles, in 48 pairs (6 + 6 + 12 + 24), biradial symmetry on the directive axis and its orthogonal axis. First cycle perfect with two pairs of the directive mesenteries attached to well-developed siphonoglyphs (2) (figure 4A-i, A-ii, A-iii). The second and third cycles bore mesenterial filaments (figure 4A-iv). Fourth cycle of mesenteries, sterile and weak (figure 4). Same number of mesenteries distally and proximally. Retractor muscles diffuse and weak (figure 4A-ii, A-iii). Ectodermal musculature, longitudinal in tentacles and radial in oral discs (figure 5C). Sphincter mesogleal, transversally stratified, strong with lamellae of mesoglea crossing from inner to outer, as the transversal stratification (figure 5, holotype NSMT-Co 1822; electronic supplementary material, figure S2, paratype CMNH-ZG 10597). Basilar muscles well developed (electronic supplementary material, figure S2E). Parietobasilar muscles, definitively weak without a penon (figure 4). Sexes dioecious, holotype female (figure 4) with mature oocytes vesicles 22.7−80.5 µm in diameter (collected in June). All the developmental stages of the ova occurred on each mesentery.

*Carcinoecium*. Thin, secreted directly under pedal disc, unremovable from pedal disc, dark brown without gloss, surface smooth without typical growth rings; coating on the gastropod shell, partly extending only to the shell aperture (figure 6A–D). No visible incorporated materials, such as fine sand or biological organisms (e.g. diatom shells) (figure 6E,F).

**Figure 7.**
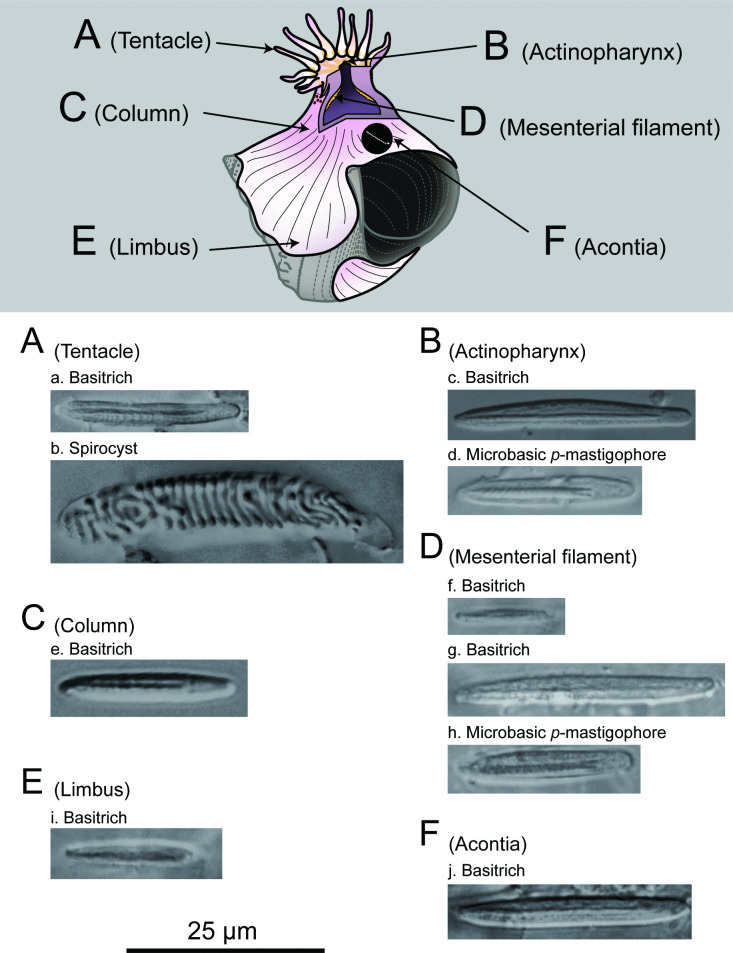
Cnidae of *Paracalliactis tsukisome* sp. nov. (holotype: NSMT-Co 1822). Positions of the sea anemone (upper diagram), including the large basitrich (A-a) and spirocyst (A*-*b) in tentacles, large (B-c) and small (B-d) basitrich in the actinopharynx, middle basitrich (C-e) in the column, microbasic *b*-mastigophore (Df) and *p*-mastigophore (D-g) in the mesenterial filament, and microbasic *b*-mastigophore (D-h) and *p*-mastigophore (E-i) in limbs, and basitrich (F-j) in the acontia.

**Figure 8.**
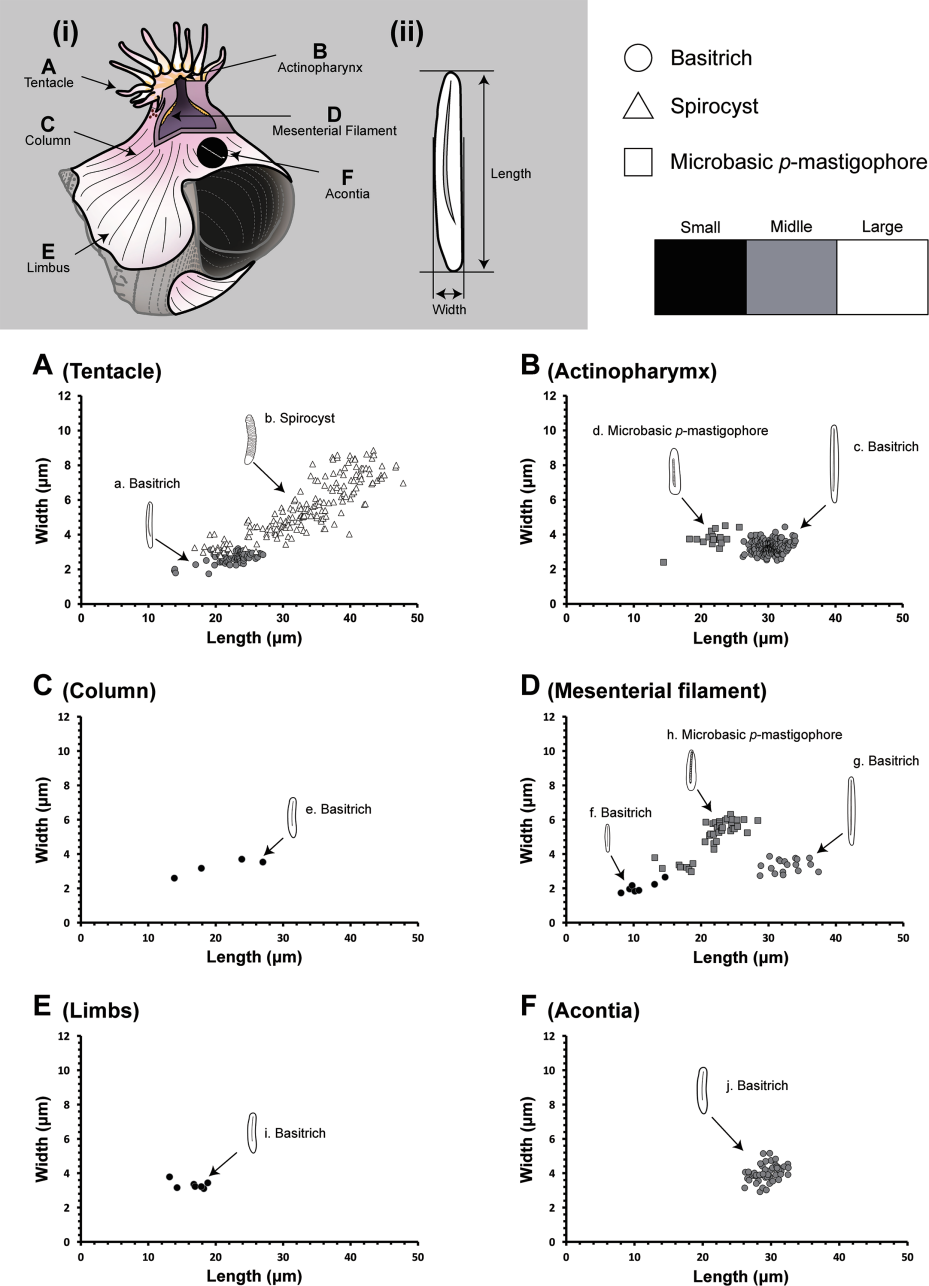
Size distribution of the *Paracalliactis tsukisome* sp. nov. cnidae (holotype: NSMT-Co 1822). (i) Examined positions of the sea anemone and the corresponding scatter diagrams (A–F). (ii) Measured parameters of the cnidae. ‘a’ to ‘j’ correspond to the examined positions indicated in figure 7.

*Cnidom*. Spirocysts in the tentacle, basitrichs in the tentacle, actinopharynx, column, mesenterial filament, limbus, and acontia, and microbasic *p*-mastigophores in the actinopharynx and mesenterial filament (figures 7 and 8, electronic supplementary material, figure S3, tables S4 and S5).

*Coloration*. Pedal disc transparent and mesenteries appearing as white line (figure 2A–D). Middle of column is translucent white, internal structures visible as pearl pink (figure 2A–D). Scapus pink/pinkish orange and white. Tentacles pinkish transparent with two orange spots near the base of each. Oral disc white, with five or six wide orange bands radially extending from mouth to tentacles. Mouth (lips, actinopharynx and siphonoglyphs) light orange (figure 2A, electronic supplementary material, video S1).

*Distribution and habitat*. The samples were collected from the Pacific side of the Sea of Kumano and Suruga Bay, facing the middle of Honshu Island, Japan. The specimens were distributed 192−470 m from the fine sand and mud (figure 1, electronic supplementary material, table S1).

*Ecological note. Paracalliactis tsukisome* sp. nov. were exclusively found on shells inhabited by *O. monstrosus*. However, *O. monstrosus* without *P. tsukisome* sp. nov. was also collected in this study. Typically, the association is one to one; one individual attached to one shell of the host hermit crab. *Paracalliactis tsukisome* sp. nov. coated the host’s gastropod shell or partly extended to the shell aperture by producing carcinoecium (figure 6A–D).

**Figure 9.**
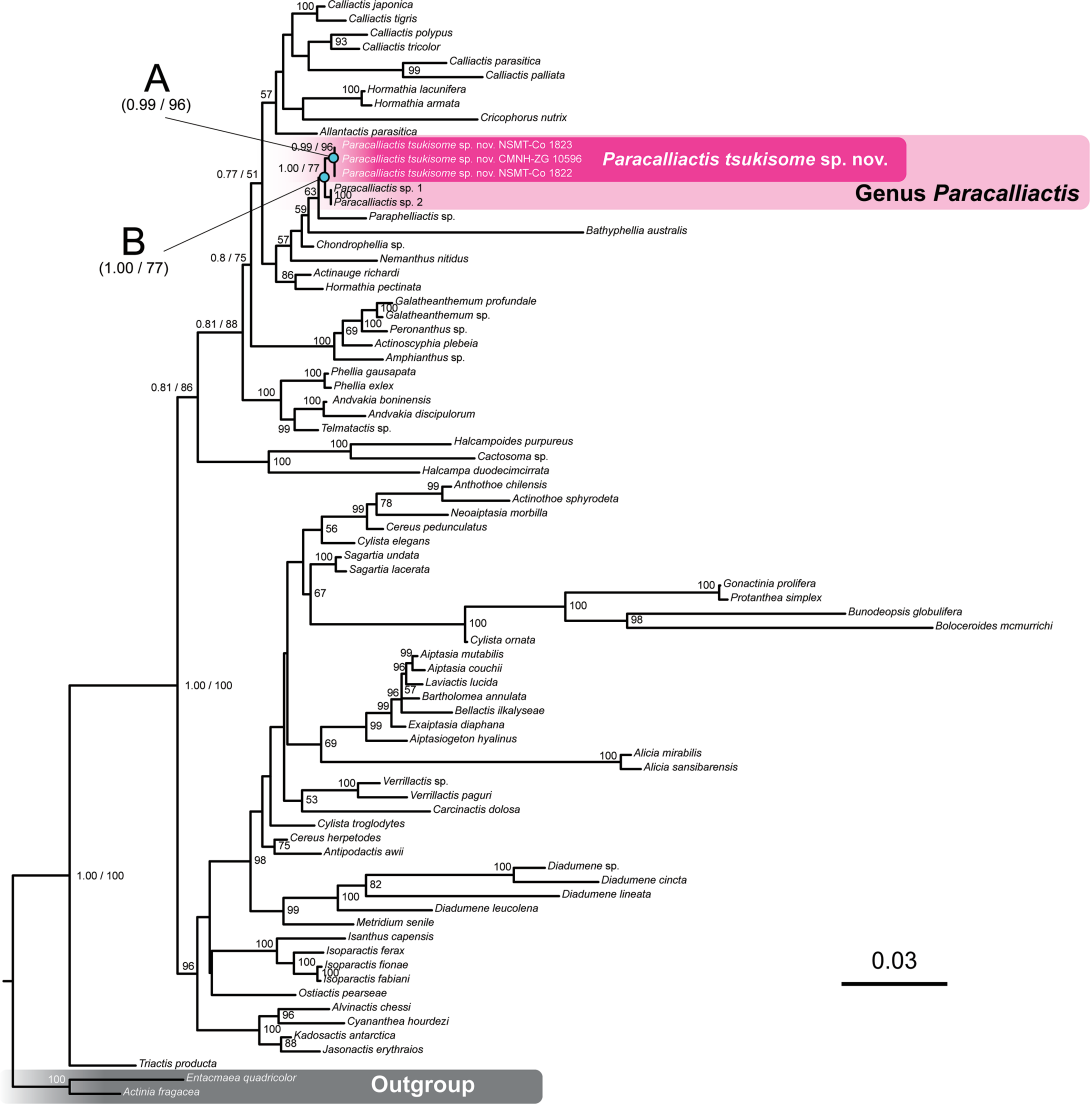
Maximum-likelihood (ML) phylogenetic tree of Metridioidea, including *Paracalliactis tsukisome* sp. nov., based on combined 18S, 28S, 16S rDNA, 12S rDNA and COXIII gene data. The numbers written with slashes above or below the branches indicate the ML bootstrap support values followed by Bayesian inference (BI) posterior probabilities of each node. Note that those without slashes only indicate the ML bootstrap support values. Red region: position of *P. tsukisome* sp. nov. Values with bootstrap support less than 50 or posterior probability = 0.50 are indicated by ‘—’. The sequences of *Actinia fragacea* (Tugwell, 1856) and *Entacmaea quadricolor* (Leuckart in Rüppell & Leuckart, 1828) (considered as an outgroup for phylogenetic analysis) were collected from GenBank (electronic supplementary material, table S2). The procedure for the molecular phylogenetic analysis generally followed that of a previous taxonomic study on CF sea anemones [13].

*Genetic analysis*. In total, 6585 bp of combined 12S rDNA, 16S rDNA, 18S rDNA, 28S rDNA and COIII genes were obtained from the holotype (NSMT-Co 1823) and two paratypes (CMNH-ZG 10596 and 10597) of the newly identified species. These sequences were compared with those of other *Metridioidea* species (GenBank; electronic supplementary material, table S2) to assess their phylogenetic relationships. The phylogenetic tree reconstructed using the five DNA markers is shown in figure 9. All *P. tsukisome* sp. nov. sequences formed a monophyletic clade supported by bootstrap values of 96% (0.99) and one posterior probability (node A of figure 9). The sister clade of *P. tsukisome* sp. nov. comprised *Paracalliactis* sp. with high support (node B) and ML bootstrap values/BI posterior probabilities of 77%/1. The interspecific variation of each sequence divergence between the two congeneric species in the phylogenetic tree was calculated as follows: 0.000 for 12S, 0.003 for 16S, 0.001 for 18S, 0.011 for 28S and 0.003 for COIII. The intraspecific variation was less than 0.000 (electronic supplementary material, table S6).

*Etymology*. The specific name ‘tsukisome’ is a classical Japanese word (桃花褐) referring to the pale pink colour of the Japanese crested ibis. It appears in an ancient Japanese poem (waka) in volume 12 of the Man'yōshū, the oldest surviving anthology of Japanese poetry. In this poem, a kimono dyed in ‘tsukisome’ symbolizes half-hearted affection, with the poet contrasting it with sincere and faithful love: ‘I won’t meet you with a half-hearted affection like the pale pink hue of a kimono’. This name aptly reflects the new species’ pale pink coloration (column and tentacles) and its species-specific symbiosis with the hermit crab, evoking a deep, faithful bond akin to that described in classical poetry.

*Taxonomic diagnosis. Paracalliactis tsukisome* sp. nov. morphologically differs from other *Paracalliactis* species in that it has four cycles of mesenteries, irregularly numbered well-developed tubercles on weak corona in the column, more than 90 tentacles, cnidae distribution on column and mesenterial filament, habitable depths of 192−470 m, distribution in the Northwest Pacific Ocean, and host species-specific associations with *O. monstrosus*.

### 3.2. Trophic interaction of the symbiotic association

**Figure 10.**
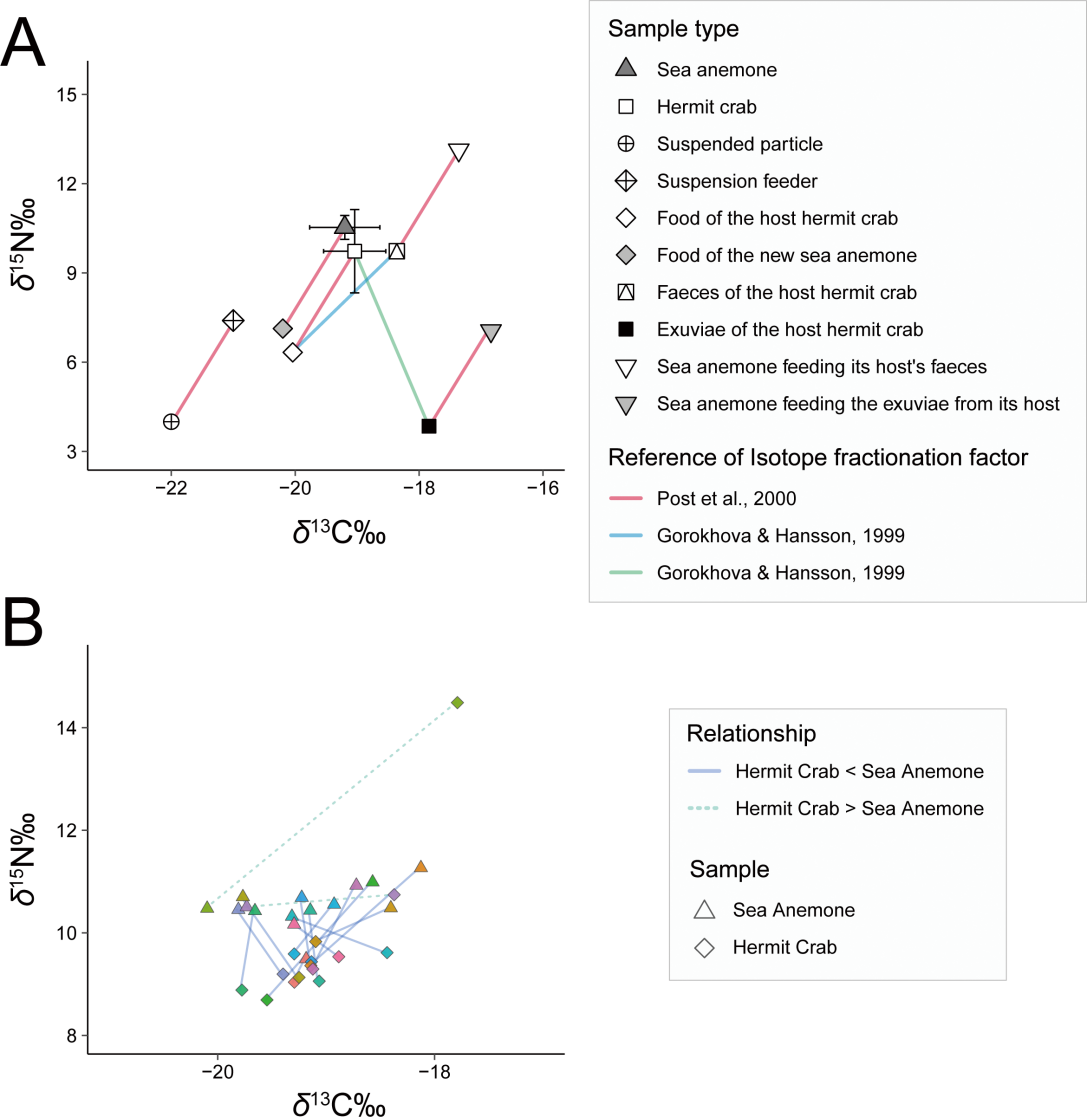
*δ*^13^C and *δ*^15^N values of (A) *Paracalliactis tsukisome* sp. nov. and its host hermit crab *Oncopagurus monstrosus* (Alcock, 1894) collected from the Sea of Kumano, off the coast of the Kumano region of the Kii Peninsula, Honshu, Japan in June 2022 (320–470 m) (present study), and estimated *δ*^13^C and *δ*^15^N values of the sediment and suspended particles collected from the nearby deep-sea station in the Sea of Kumano (150–250 m) on March 2007, as reference data [45]; (B) relationships of the *δ*^13^C and *δ*^15^N values of the symbiotic pair. The pairs connected by dotted lines indicate that the sea anemone has a higher *δ*^15^N value than its host hermit crab. The pairs connected by solid lines indicate that the host hermit crab has higher *δ*^15^N values than its associated sea anemone. The colour of each line coincides with the symbiotic pair.

The *δ*^13^C value of *P. tsukisome* sp. nov. and *O. monstrosus* ranged from −20.1‰ to −18.1‰ (average ± s.d. = −19.2 ± 0.6) and −19.8‰ to −17.8‰ (average ± s.d. = −19.0 ± 0.5), respectively, without any significant difference between them (Welch’s *t*‐test: *δ*^13^C, *t* = −0.73, d.f. = 27.78, *p* = 0.47). The *δ*^15^N value of *P. tsukisome* sp. nov. and *O. monstrosus* ranged from +9.5‰ to +11.4‰ (average ± s.d. = +10.5 ± 0.4) and +8.7 to +14.5 (average ± s.d. = +9.7 ± 1.4), respectively. Moreover, each *δ*^15^N value was significantly separated (Welch’s *t*‐test: *δ*^15^N, *t* = 2.33, d.f. = 17.8, *p* < 0.05), and the value of *P. tsukisome* sp. nov. was approximately 0.8‰ higher than that of *O. monstrosus* (figure 10, electronic supplementary material, table S7). The *δ*^13^C values of both sea anemones and hermit crabs were 2−4‰ higher than the estimated value of suspended particles from the region (−22‰ [45]). The *δ*^15^N values of anemones and hermit crab were approximately 5−7‰ higher than the estimated value of suspended particles from the region (+3 to +5‰ [45]).

### 3.3. Three-dimensional investigation of the attachment direction

**Figure 11.**
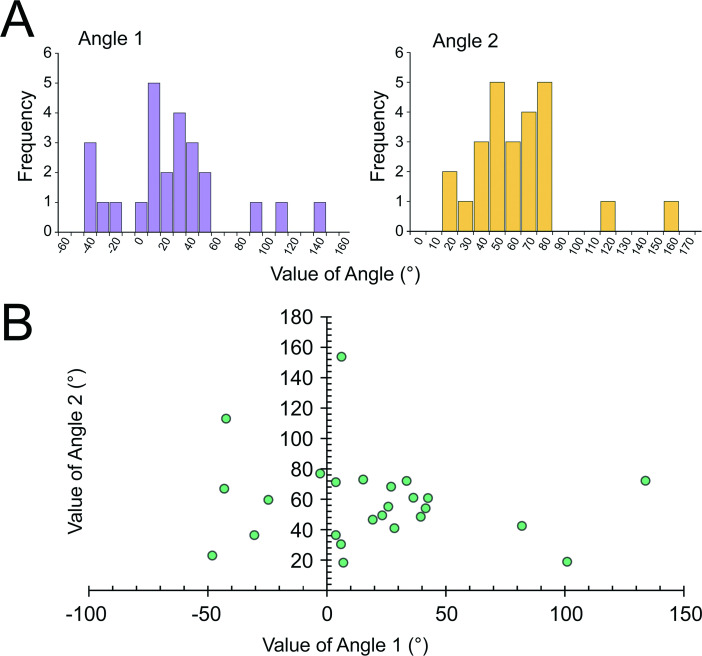
Attached position of the *Paracalliactis tsukisome* sp. nov. on its host gastropod snail shells. (A) Frequency of the value of Angles 1 and 2. (B) Relationship of the value of Angles 1 and 2. Twenty-five individual specimens, which were collected from the Sea of Kumano, off the coast of the Kumano region of the Kii Peninsula, Honshu, Japan, in July 2022 were examined.

Twenty-five fixed specimens of *P. tsukisome* sp. nov. were scanned and analysed three-dimensionally to determine the attachment direction. The mean ± s.d. of Angle 1 is = 19.38° ± 41.95° (maximum = 133.9° and minimum = −43.13°), and that of Angle 2 is 57.93° ± 28.60° (maximum = 153.74° and minimum = 18.22°). The results of the three-dimensional investigations are presented in electronic supplementary material, table S8; Figure 11 shows the relationships of the attached position of *P. tsukisome* sp. nov. on the host gastropod snail shells.

### 3.4. Size comparison of *Oncopagurus monstrosus* and other *Oncopagurus* species

**Figure 12.**
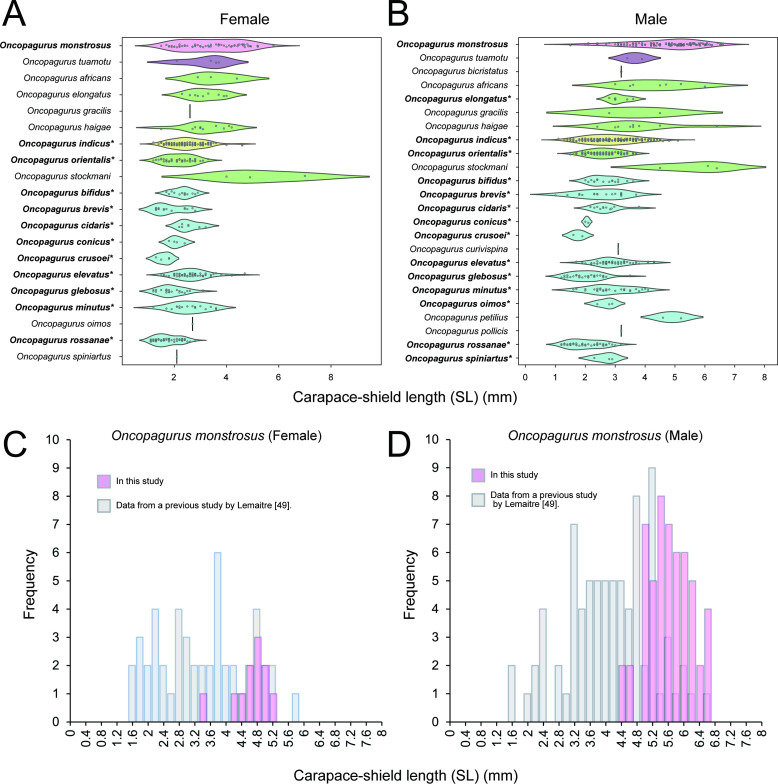
Size differences of the measured carapace-shield length (SL) in *Oncopagurus* species. Violin plots illustrate the SL of females (A) and males (B) of *Oncopagurus* species. *Oncopagurus monstrosus* (Alcock, 1894) is represented by the pink boxplot. Each species is categorized by their symbiotic style as follows: red, CF but opportunistic (anthozoan polyps); purple, species-specific (anthozoan polyps); green, opportunistic (anthozoan polyps or Hydractinia colonies); yellow, the sympatric species of *O. monstrosus, Oncopagurus indicus*, opportunistic (anthozoan polyps or Hydractinia colonies); blue, non-symbiotic species. The plots represent females (A) and males (B). Asterisks (*) indicate species with statistically significant differences from *O. monstrosus* based on Welch’s *t*‐test with Bonferroni adjustments. Histograms illustrate the size differences between examined specimens of *O. monstrosus* in this study definitively associated with *P. tsukisome* sp. nov., and data from Lemaitre [49] previous study possibly related to actiniarian species. The graphs represent females (C) and males (D).

A comparison of the size and frequency distributions of 23 *Oncopagurus* species, including the sympatric species *O. indicus* and those of *O. monstrosus*, are compared in figure 12A,B and electronic supplementary material, figure S4A,B. The number of specimens used for these comparisons and the body size metrics (mean, minimum, median and maximum values) for each species are summarized in electronic supplementary material, table S9.

Significant differences in body size were observed between *O. monstrosus* and 10 *Oncopagurus* species (*O. bifidus*, *O. brevis*, *O. conicus*, *O. curvispina*, *O. glebosus*, *O. indicus*, *O. oimos*, *O. orientalis*, *O. rossanae* and *O. spiniartus*) for both sexes. Compared with those in five species (*O. cidaris*, *O. crusoei*, *O. elevatus*, *O. elongatus* and *O. minutus*), significant differences were observed only in males but not in non-ovigerous females and/or single ovigerous females. Furthermore, no significant differences were observed between six species (*O. africanus*, *O. gracilis*, *O. haigae*, *O. petilus*, *O. stockman* and *O. tuamotu*) in either sex (electronic supplementary material, table S9).

The body size of examined specimens of *O. monstrosus* with a confirmed association to *P. tsukisome* sp. nov. was significantly larger than that of specimens reported in a previous study by Lemaitre [49], which were probably associated with an actiniarian species (all females: *t* = 6.4, d.f. = 53.71, *p* ≤ 0.01; all males: *t* = 9.73, d.f. = 123.38, *p* ≤ 0.01; non-ovigerous females: *t* = 5.0, d.f. = 39.15, *p* ≤ 0.01; ovigerous females: *t* = 5.1, d.f. = 14.36, *p* ≤ 0.01). The mean body size (± standard deviation, s.d.) of each group was as follows: all females, this study, 4.55 ± 0.44 mm; Lemaitre [49], 3.27 ± 1.10 mm; all males, this study, 5.49 ± 0.56 mm; Lemaitre [49], 4.08 ± 1.11 mm; non-ovigerous females, this study, 4.51 ± 0.49 mm; Lemaitre [49], 3.25 ± 1.17 mm; ovigerous females, this study, 4.71 ± 0.12 mm; Lemaitre [49], 3.34 ± 0.97 mm (figure 12C,D,4 and electronic supplementary material, figure S4C,D).

## 4. Discussion

### 4.1. Differential diagnosis of *Paracalliactis tsukisome* sp. nov

This study scientifically describes *P. tsukisome* sp. nov. (Cnidaria: Actiniaria: Hormathiidae), a species-specific hermit crab-associated sea anemone. The molecular analysis identified the new species as a *Paracalliactis* species in the phylogenetic tree that morphologically differs from other *Paracalliactis* species (see multi-access keys in table 1 for comparison with other *Paracalliactis* species). By contrast to previously described *Paracalliactis* species, *P. tsukisome* sp. nov. is distributed at relatively shallow depths in the deep sea and is often caught by deep-sea trawl nets at approximately 200−500 m in the Pacific Ocean facing the middle of Honshu Island, Japan. Therefore, this species could be a variable model for understanding how *Paracalliactis* species or specific hermit crabs find a partner and continue species-specific symbiotic relationships in the deep sea.

**Table 1.** Multi-access keys for *Paracalliactis* species based on morphology, biogeography and symbiotic association with a hermit crab. ‘–’ indicates that no records were obtained from a previous study.

			*Paracalliactis tsukisome* sp. nov.	*P*. *azorica*	*P. consors*	*P*. *involvens*	*P. michaelsarsi*	*Paracalliactis* sp. (ms. n, described in Gusmão [9] as *P*. *niwa*)	*P*. *obvolva*	*P*. *rosea*	*P*. *sinica*	*P*. *stephensoni*	*P. valdiviae*
morphology	direction of oral disc (position on the shell)		face upward (dorsal side of the host hermit crab)	oriented towards substrate (ventral side of the host hermit crab)	face upward (dorsal side of the host hermit crab)	face upward (dorsal side of the host hermit crab)	both	face upward, opposite side of the gastropod shell aperture	face upward (dorsal side of the host hermit crab)	face upward (dorsal side of the host hermit crab)	face upward (dorsal side of the host hermit crab)	face upward (dorsal side of the host hermit crab)	face upward (dorsal side of the host hermit crab)
	cycles of mesenteries		4	5	3−4	4	4	5	5	4	5	—	4
	scapus and scapulus		unclearly divided	divided, reduced and strongly introverted scapulus	well divided, thin cuticle is only in the scapus	well divided, the scapus is covered with a thin cuticle	well divided	well divided	divided, short scapulus	well divided			
	distribution of tubercles		irregularly distributed	—	developed, sometimes not easily identified	without tubercles	without tubercles	without tubercles	—	well-developed, the appearance of tubercles present in the distal part of the scapus varies	tubercles on the distal part of scapus	conspicuous but wide variability, depends on the development	well developed
	status of tubercles		some of tubercles are covered by a thin, brown cuticle	—	—	—	—	lacks tubercles in the distal most part of the scapus	—	some of tubercles are covered by a thin and frail brown cuticle	—	tubercle development varies	some of tubercles are covered by a thin, brown cuticle
	corona of tubercles		incomplete	—	complete	—	—	—	—	complete	—	complete	complete
	number of tentacles		90−95	—	60−96	96?	96	up to 172	190−250	up to 96	192−394	—	90−96?
cnidom	tentacle	size range of basitrich	1	2	1	1 or 2	2	1	1	2	2	1	1
		size range of spirocyst	1	—	1	1	1	2	2	1	1	1	2
		microbasic *b*-mastigophore or *p*-mastigophore	—	—	*b*-mastigophore	—	—	—	*b*-mastigophore	*p*-mastigophore	—	—	—
	actinopharynx	size range of basitrich	1	1	1	1	—	1	1	1	1	1	2
		size range of spirocyst	—	—	—	—	—	—	—	—	1	—	—
		microbasic *b*-mastigophore or *p*-mastigophore	*p*-mastigophore	—	*p*-mastigophore	*p*-mastigophore	*p*-mastigophore	*p*-mastigophore	*p*-mastigophore	*p*-mastigophore	—	*p*-mastigophore	—
		size range of basitrich of *p*-mastigophore	1		1	1		2					
	column	size range of basitrich	1	—	2	2	1	2	2	2	1	2	—
		microbasic *b*-mastigophore or *p*-mastigophore	—	—	*p*-mastigophore	*p*-mastigophore	*p*-mastigophore	*p*-mastigophore	*p*-mastigophore	*p*-mastigophore			
	mesenteril filament	size range of basitrich	2	1	2	2	1	1	2	2	1	1	—
		size range of spirocyst	—	—	—	—	—	—	—	—	1	—	—
		microbasic *b*-mastigophore or *p*-mastigophore	*p*-mastigophore	*p*-mastigophore	*p*-mastigophore	*p-*mastigophore	*p*-mastigophore	*p*-mastigophore	*p*-mastigophore	*p*-mastigophore	—	*p*-mastigophore	—
	acontia	size range of basitrichs	1	1	2	1 or 2	1	1	2	2	1	2	1
coloration	tentacles		pinkish transparent with two orange spots near the base of each tentacle	—	light beige	salmon colour, bright reddish orange	reddish dark pink, white, beige, pink	tentacles are dark white or transparent	—	pale pink, rosy pink to a light salmon orange, apricot. No bars, bands, or other marks on the tentacles.	—	—	white, light pink
	mouth		orange	—	—	—	—	—	—	—	—	—	—
	oral disc		white, with the 5 or 6 orange wide band which are radially extending from the mouth to the tentacles	—	light beige	—	colourless?	circular, with white striations corresponding to the mesenterial insertions	—	transparent/translucent, the pinkish orange throat and the bright orange endodermal lining of the throat are visible through the oral disc, or opaque white	lighter than column, white, not shiny	—	—
	oral disc		white, with the 5 or 6 orange wide band which are radially extending from the mouth to the tentacles	—	light beige	—	colourless?	circular, with white striations corresponding to the mesenterial insertions	—	transparent/translucent, the pinkish orange throat and the bright orange endodermal lining of the throat are visible through the oral disc, or opaque white	lighter than column, white, not shiny	—	—
	scapus		pink or pinkish orange	light brown or slightly pinkish, translucent in middle part	—		pale brown or flesh pink	mostly brown with longitudinal darker brown stripes	—	pink or rosy pink	—	pale yellow, flesh pink or white, except in the basal region where it is translucent (the mesenteries are then visible by transparency)	—
	scapulus		white	—	—	rose orange	—		—	pink or rosy pink	—	dark pink, with deeply striated longitudinally	—
	column		translucently white, the internal structures visible as pearl pink	—	—		—	light brown with deep longitudinal furrows that coincide with the scapus's coloration pattern	bright pink, with slightly darker longitudinal stripes	white	longitudinal line appearing as dark bands, and 24 coarse dark stripe on the column	reddish dark pink at the border between scapus and scapulus	—
	pedal disc		transparent, the mesenteries and mesenteril filament appearing as white line on the pedal disc	—	—	rose orange	—	light brown, transparent. Mesenterial insertions are represented by dark lines that can be seen through the pedal disc (and the column).	—	white or light beige	—	—	pink or transparent
distribution and habitat	type locality or distribution		Sea of Kumano and Suruga Bay, Pacific side facing the middle of Honshu Island, Japan (the northwestern Pacific Ocean)	off the coast of Azores, the North Atlantic Ocean	The western Atlantic Ocean (eastern United States), the eastern Pacific Ocean (Galapagos Islands, Ecuador)	The eastern Pacific Ocean	The North Atlantic Ocean	Tasman Sea off the western coast of New Zealand, the western Pacific Ocean	Gulf of Mexico, the North Atlantic Ocean	Taiaroa Canyon, off the east coast of New Zealand, the western Pacific Ocean	East China Sea, the Northwestern Pacific Ocean	The west coast of Ireland; the North Atlantic Ocean	The coast of Somaliland in east Africa, Indian Ocean
	depth		192−470 m	2900−3800 m	600−660 m	2638−2755 m	4166−4700 m	2417−2421 m	375−550 m	approx. 50−3,000 m	39−47 m		
	substrate		fine sand, mud	—	—	—	—		—	—	sandy mud, muddy sand	—	—
host association	no. of the recorded host species		1	—	1		—	—	1	5	—	2	2
	host hermit crab species		*Oncopagurus monstrosus*	unidentified	no host hermit crab was preserved with syntypes, *Sympagurus pictus*?	unidentified	unidentified	unidentified	*Sympagurus pictus*	*Diacanthurus rubricatus*; *Leptomithrax longipe*s; *Lophopagurus* (*Lophopagurus*) *lacertosus*; *Paguristes subpilosus*; *Sympagurus dimorphus*	unidentified	*Parapagurus pilosimanus; Parapagurus nudus*	*Oncopagurus bicristatus; Sympagurus andersoni*
	carcinoecium		very thin, but enlarge the host snail shells	light yellow with brown stripe	thickened closed to the limbs, but not enlarge the host snail shells	enlarge the host snail shells	bronze coloration, enlarge the host snail shells	thin, fragile, bronze cuticle, not extend beyond the aperture of the shell and not enlarge the living space of the hermit crab	enlarge the host snail shells	secretes a cuticle, not enlarge the host snail shells	no cuticle or carcinoecium	enlarge the host snail shells	very thin darker golden colour cuticle, sometime absent, does not enlarge the host snail shells
references			(in this study)	[53]	[8,9]	[8,9,56–58]	[9,53,59]	[9]	[8,9]	[9,23,24,60]	[61]	[9,53,59,62]	[9,63]

The present study constitutes the third photographic record of this genus and the first record containing species-identified specimens. To our knowledge, living specimen records are extremely limited. Most previous records included photographs or illustrations of fixed specimens of *Paracalliactis* species. Living records have only been provided in the photographs depicted in figure 1F,G in a paper by Gusmão *et al.* [13] and figure 5F in another paper by Sanamyan *et al.* [56]. Gusmão *et al.* [15] identified the photographed specimens as *Paracalliactis* sp., whereas Sanamyan *et al.* [56] identified them as *P*. cf. *involvens*.

Herein, a living specimen of *P. tsukisome* sp. nov. was also successfully recorded through videography (figure 2A–D, electronic supplementary material, video S1). The video shows that this new species possesses two orange spots at the base of each tentacle, thus exhibiting one of the conspicuous characteristics that may easily distinguish it from at least six *Paracalliactis* species (see coloration in table 1). Observation of the mouth, tentacles and oral disc of living *Paracalliactis* species is particularly difficult, as live specimens fully retract their mouth soon after collection and rarely relax again in captive conditions. Thus, records showing their living states will be valuable information for avoiding the taxonomical confusion surrounding the group of *Paracalliactis* species and helping to identify the species, especially in a field survey. In some *Actiniaria* species, distinctive differences in coloration have been reported between closely related species to reliably distinguish them in the field [64], yet in other genera, coloration is not indicative and therefore represent cryptic species e.g. *Actinia* spp [65]. It is still to be confirmed if coloration is a suitable diagnostic feature to distinguish *P. tsukisome* sp. nov. from other *Paracalliactis,* as *in vivo* information is lacking for many species (*P. azorica, P. obvolve, P sinica, P. stephensoni*). Body coloration in animals serves various functions, including camouflage [66], social communication [67,68], mimicry [69] and thermoregulation [70,71], and ecological selective pressures can produce various colour morphs of the same species. Coloration varies substantially correlating to the substrate colour, environmental conditions and season, thereby serving as camouflage against visual predators [72,73]. The present description, together with future studies, may provide new insights into the adaptations and effects of natural selection on animal body coloration in the deep-sea benthic environment, where *Paracalliactis* species occur, as well as into the speciation processes within this genus.

Moreover, considering the successful observation of living specimens through video recording, it was hypothesized that seawater temperatures of approximately 10°C are suitable for the long-term rearing of this species, at least for approximately month (the period before and after current behavioural observation). The taxonomic confusion mentioned earlier was probably exacerbated by the lack of opportunities to observe well-preserved species due to difficulties in sample collection and preservation. Therefore, this information on the rearing condition is also valuable not only for future ecological, behavioural and evolutionary studies on maintaining species-specific interactions and mechanisms of shell-forming ability but also for preparing specimens for taxonomical study.

### 4.2. Taxonomic confusion between *Paracalliactis* species

Some taxonomic confusion surrounds the *Paracalliactis* species. Due to the paucity of knowledge regarding their morphological characteristics and insufficient or fragmented morphological descriptions, differentiating between *Paracalliactis* species can be challenging [9]. Accordingly, in this study, some taxonomically confused specimens were also included when comparing *P. tsukisome* sp. nov.

For instance, although the taxonomic status of *P. involvens* is still unclear, this species was included in the species comparison. Sanamyan *et al.* [56] reported *Paracalliactis* cf. *involvens* as being synonymous with the *Paracalliactis consors* reported by Daly *et al.* [8], from the Western Bering Sea, Northwest Pacific Ocean. Sanamyan *et al.* also noted morphological differences between *P. involvens* and *P. consors*, namely the absence of tubercles in *P. involvens*, highlighting the species variability of *P. involvens* [56]. Additionally, the depth at which the species were collected differed between these two species. Specifically, *P. involvens* are collected from a depth of 2638−2755 m, while the holotype specimen is collected from 1360 m. By contrast, *P. consors* are recorded from 600−660 m [9]. Thus, considering the species variability of *P. involvens*, it was included in the species comparison of this study.

Two *Paracalliactis* species, *P. rosea* [60] and *P. sinica* [61], also have taxonomical confusion, that is, the possibility of the existence of cryptic species distributed in shallower depth ranges. Although most of the *Paracalliactis* species have been recorded in the deep sea, some individuals of these two species were collected from shallow to deep depths; *P. rosea* is collected from the range of 50−3000 m depth [9,23,24] and *P. sinica* is collected from shallow depths of 39−40 m [61]. It is, therefore, possible that some undescribed species are included under the name of these species. Moreover, specific characteristics of *P. sinica*, such as five mesentery cycles, well-developed parietobasilar muscle, lack of microbasic *p*-mastigophores in the column, and absence of cuticle or carcinoecium, are shared with species belonging to the genus *Calliactis*. Accordingly, Gusmão emphasized the necessity of re-examining the material type of *P. sinica* and the existence of its cinclides to clarify its taxonomic status [9]. In this study, however, these two species were assigned as *Paracalliactis* species and included in species comparison.

Although the taxonomic status of *P. niwa* (ms. n. [9]) is also controversial, this species was included in species comparison because it is morphologically distinct from the new species and other *Paracalliactis* species. However, the taxonomic status of this species is uncertain as it has only been described in Gusmão’s dissertation [9] and was not included in the taxonomic description of *Paracalliactis* genera ‘*Included species*’ in Gusmão’s subsequent studies [55] or in the summarized species list in the World Register of Marine Species (WoRMS) database; the validity of each species identification was checked by referring to their nomenclature in the WoRMS database (accessed on 5 January 2025). Thus, the taxonomic status of *P. niwa* should be resolved to elucidate the species diversity of *Paracalliactis* genera in the western Pacific Ocean. Nevertheless, *P. niwa* was included in the species comparison in this study as *Paracalliactis* species (ms. n, described by Gusmão [9] as *P. niwa*) (table 1 and electronic supplementary material, table S5).

### 4.3. Nematocyst nomenclature of *Paracalliactis* species

The distribution and size ranges of cnidae in *P. sinica* and *P. valdiviae* should be re-examined to allow more precise comparisons and to avoid taxonomic confusion. The inconsistencies related to nematocyst classifications may exacerbate taxonomic confusion. For example, Carlgren used the presence or absence of different types in the acontia to define families [19]; however, the number of nematocyst types in the acontia can vary, even within species of the same genus [12,13,74,75]. Moreover, nematocyst classification is complex, with several non-standardized nomenclature systems [33,61,63,76–79] with mismatched classifications currently being used, impeding accurate comparisons among studies [75,80]. Some mismatched cnidae classifications were also found in the original description of *P. sinica* and *P. valdivia*; the length data of these classifications were provisionally assigned to the classification that is known as the cnidae-characteristics of *Paracalliactis* species. Furthermore, although Carlgren [62] noted one size range of basitrichs in the acontia of *P. stephensoni*, Doumenc [53] updated it as two size ranges of basitrichs. The present study, therefore, follows Doumenc’s [53] description as it represents the latest information on *P. stephensoni* (table 1). In the current study, the diagnostic characteristics of the poorly described genus were updated based on the description of a new species, to further avoid taxonomic confusion (table 1, electronic supplementary material, tables S4 and S5).

### 4.4. Symbiotic association

*Paracalliactis tsukisome* sp. nov. is the second species of this genus with a species-specific symbiotic relationship with one hermit crab species on the deep-sea floor. Although all previously reported *Paracalliactis* species have symbiotic relationships with hermit crabs living on the deep-sea floor, only two species, *P. consors* and *P. obvolva*, are recorded from the snail shell inhabited by *Sympagurus pictus* Smith, 1883 [8,9]. However, no host hermit crab was preserved with syntypes of *P. consors*, and the number of observed specimens remains unknown; whether these species have species-specific symbiotic relationships or not should be studied further [8,9]. In the present study, *P. tsukisome* sp. nov. was exclusively found on shells inhabited by *O. monstrosus*. Moreover, *O. monstrosus* was also identified as a species that matched the description by Lemaitre, i.e. ‘*Gastropod shells usually with actinian attached to shell*’ [49]. On this basis it is reasonable to conclude that *P. tsukisome* sp. nov. has a species-specific or an obligate symbiotic relationship with *O. monstrosus* in the northwestern Pacific Ocean, off the Pacific coast of central Honshu, Japan. The host hermit crab *O. monstrosus*, however, has a broader distribution across the Indo-West Pacific region and depth range (188−1000 m) [49], therefore *P. tsukisome* sp. nov. may potentially occur throughout the Indo-West Pacific and at greater depths. This is further discussed in §4.5.

Among other *Paracalliactis* species, *Paracalliactis stephensoni* has symbiotic relationships with two *Parapagurus* species, *Parapagurus pilosimanus* (Smith, 1879) and *Parapagurus nudus* (A. Milne-Edwards, 1891). Meanwhile, *P. valdiviae* exhibits symbiotic relationships with *O. bicristatus* and *Sympagurus andersoni* (Doumenc, 1975). *Paracalliactis rosea* is commonly collected with three hermit crab species, namely *Lophopagurus* (*Lophopagurus*) *lacertosus* (Henderson, 1888), *Diacanthurus rubricatus* (Henderson, 1888), *Paguristes subpilosus* (Henderson, 1888) and *Sympagurus dimorphus* (Studer, 1883) and from one spider crab *Leptomithrax longipes* (GM Thomson, 1902). In shallow waters (approx. 50−100 m), *P. rosea* is commonly associated with *D. rubricatus*, *P. subpilosus* and *L. longipes*, while deep-sea individuals (400–1000 m) have only been recorded on hermit crab shells used by *S. dimorphus* [60]. By contrast, the symbiotic relationships of *P. azorica*, *P. michaelsarsi*, *P. niwa* (ms. n. [9]) and *P. sinica* are uncertain.

In summary, the specific relationships of *P. tsukisome* sp. nov. are rare among the *Paracalliactis* species. Given its collectability and manageable rearing conditions, this species could serve as a model organism for investigating the ecological mechanisms underlying the maintenance of these symbiotic relationships in natural environments.

### 4.5. Host distribution

*Paracalliactis tsukisome* sp. nov. may have a broader distribution across the Indo-West Pacific region, given the extensive range of *O. monstrosus* in these oceans. While the newly described species has been described based on specimens found on the snail shells of *O. monstrosus* collected from the Pacific coast of central Honshu Island, Japan, further investigations are needed to confirm its presence across the host’s distribution range.

The host hermit crab, *O. monstrosus,* is distributed at 188−1000 m in the Indo-Pacific Ocean (Gulf of Aden, Bay of Bengal, Japan, Taiwan, Philippines, Indonesia, Solomon Islands, Tonga Islands, New Caledonia and Australia) [49,50,81]. In Japan, in addition to the sampling sites of the present study, *O. monstrosus* has been recorded on the Kii Channel off Hiwasa in Tokushima Prefecture, Tosa Bay off the coast of Kouchi Prefecture, Hyuganada off the coast of Miyazaki Prefecture and the East China Sea near Kagoshima Prefecture [82–85]. Moreover, *O. monstrosus* has been found in gastropod shells with sea anemone species. For instance, Henderson stated, ‘*A large number of specimens, the majority inhabiting Rostellaria shells, some of which have an investing Hpizoanthus, others an Actinia*’ when describing *O. monstrosus* collected from the Madras Coast at a depth of 265−457 m [86]. In 1901, Alcock reported the collection of *O. monstrosus* associated with the sea anemone from the Bay of Bengal at a depth of 265−521 m, the Andaman Sea at a depth of 740 m, and the Arabian Sea, off Ceylon and the Malabar coast at a depth of 259−849 m [87]. In 1912, Kemp and Sewell also collected six *O. monstrosus* specimens from the Laccadive Sea, southwest of Karnataka at 433 m, and stated that most of the shells were encrusted by an anemone [88]. McLaughlin *et al*. also mentioned that *O. monstrosus* collected off the southwest coast of Phuket, Thailand and the northeast coast of Taipei, respectively, were found in gastropod snail shells inhabited by a sea anemone [89,90].

Therefore, if *P. tsukisome* sp. nov proves to be an exclusive species-specific or in an obligate symbiotic relationship with *O. monstrosus*, the species distribution may be covering a wider range than already documented for the Indo-Pacific Ocean and include near the Arabian Sea, Bay of Bengal, to the East China Sea. Further research on the distribution and genetic differentiation of *P. tsukisome* sp. nov and its host hermit crab in the vast Indo-West Pacific region is required to elucidate the evolution and maintenance mechanism of host species-specific interactions.

### 4.6. Trophic interaction of the symbiotic association

The current isotope characteristics suggest that *P. tsukisome* sp. nov. consumes the faeces of the host as scavengers/detritivores as well as the suspended particles from the surrounding environment (figure 10). The different *δ*^15^N values between the crab and the anemone strongly imply that *P. tsukisome* sp. nov. do not solely depend on the food residue of the host hermit crab. Large deviation from the isotopic compositions of suspended particles strongly indicate that these particles are not the sole food source, considering that the enrichment is much larger than that expected from the general trophic enrichment factors at +1‰ and +3.4 ‰ for *δ*^13^C and *δ*^15^N, respectively (e.g. Post *et al.* [91]). To date, only one study has reported isotope fractionation between the faeces and the diet in crustaceans (*Mysis mixta* Liljeborg, 1853) with the faecal *δ*^13^C and *δ*^15^N values being +1.4‰ and +3.4‰ higher than the diet values [92]. Thus, the isotope *δ*^13^C and *δ*^15^N values of a consumer of hermit crab faeces are estimated to be −17.64‰ and 13.13‰ for *δ*^13^C and *δ*^15^N, respectively, based on the average isotope values of hermit crab and general trophic enrichment factors (*δ*^13^C = −19.04‰, −1‰ (trophic fractionation, diet) +1.4‰ (faeces fractionation) +1‰ (trophic fractionation, consumption) = −17.64‰ and *δ*^15^N = +9.73‰, −3.4‰ (trophic fractionation, diet) + 3.4‰ (faeces fractionation) + 3.4‰ (trophic fractionation, consumption) = + 13.13‰).

The isotope values of the anemone (*δ*^13^C = −19.2‰ and *δ*^15^N = +10.5‰) are around the median value of the two food components (plus one trophic enrichment), namely suspended particles (*δ*^13^C = −21‰ and *δ*^15^N = +7.4‰) and faeces of hermit crab (*δ*^13^C = −18.64 and *δ*^15^N = +9.73). It should be emphasized that the isotopic discrimination factor between food and faecal material is not well understood [46]; therefore, the quantitative evaluation of the relative contribution of food source is very difficult without the precise evaluation of isotope discrimination.

The exuviae may not be a significant dietary source for the new sea anemone, possibly because of the low frequency of moulting. The expected isotopic values of the anemone, based solely on the hermit crab exuviae and calculated using the isotope fractionation factor of *M. mixta* [93], are notably low (δ^13^C = −16.84 and δ^15^N = +7.25). These values, however, do not correspond to the observed isotopic signatures of the anemone, suggesting alternative or different dietary sources.

Although two symbiotic pairs of hermit crabs have demonstrated irregularly higher *δ*^13^C values than sea anemones did (figure 10B), this may be attributed to hermit crabs being omnivorous and able to move to explore food; hence, these hermit crab individuals may have been examined just after they fed on different food sources, which may not have been reflected in the *δ*^13^C and *δ*^15^N values of the sea anemone. Therefore, it is reasonable to conclude that *P. tsukisome* sp. nov. uses the host as the food resource on the deep-sea floor.

Although the feeding habits of the hermit crab associated with sea anemones remain poorly studied, *Calliactis parasitica* (Couch, 1842) and *S. calcifer*, symbiotic sea anemones are thought to obtain more food from seawater, sediment surfaces or food residuals of the host hermit crab shells. *Calliactis parasitica* is a suspension feeder, suggested based on the coelenteron content that exploits the mobility of hermit crabs to increase their feeding potential [27]. Moreover, *C. parasitica* is attached to the point where its tentacle reaches the benthic substrate, which may take advantage of organisms deposited as food [26,27]. Behavioural observations of *S. calcifer* in aquariums also suggest that they potentially feed on suspended particulate organic matter from the water column or food residuals of hermit crabs [12]. However, these assumptions are based on circumstantial evidence, such as the daily observations of hermit crab behaviour and motility, with no quantitative evidence to support the hypothesis [25].

Therefore, our results provide evidence of the benefit incurred by the sea anemone: the newly identified sea anemone may participate in unidirectional CF as compensation for using the host as a food resource on the deep-sea floor.

### 4.7. Unidirectional attachment of the *Paracalliactis tsukisome* sp. nov

The present study indicated that the actinopharyngeal position of *P. tsukisome* sp. nov. has a unidirectional trend on the host gastropod shell. The ecological/evolutional significance of this unidirectional movement should be discussed further. However, considering the CF ability and trophic interaction of *P. tsukisome* sp. nov. with its host hermit crab, this behavioural trend might be evolved related to the CF process and/or feeding behaviour.

The CF ability is an especially remarkable evolution in biradial sea anemones because unidirectional elongation with the growth rate gradient along the aperture at a constant angle and shape is essential for forming a spiral gastropod shell [17]. If their unidirectional movement relates to their CF ability, *P. tsukisome* sp. nov. may possess carcinoecium-producing organs bilaterally or non-uniformly below the actinopharyngeal position in the pedal disc or may recognize the shell aperture’s direction (or hermit crab’s head direction) by some physical stimulation from the water current or hermit crab.

Given that the shape of the carcinoecium varies among species and genera, the forward direction for constructing the shell shape may also differ according to species. For example, the carcinoecium secreted by *Paracalliactis* spp. is a tube-like structure that differs from the typical shape of a gastropod shell, as observed in *Stylobates aeneus* [22,94]. Other *Stylobates* species, such as *S. loisetteae* and *S. calcifer*, produce a dome-shaped carcinoecium rather than a real gastropod-shaped carcinoecium [12,94]. Moreover, the direction of carcinoecium elongation may be related to the snail shell shape as well as the state of the host hermit crab, such as the growth stage, sex, size and walking direction. Therefore, in the future, it will be necessary to compare the relationship between the position of the sea anemone and the direction of the host hermit depending on these conditions, with a focus on species/genus differences, to understand whether the unidirectional movement evolved multiple times independently or convergently, in the *Paracalliactis* species and other CF sea anemones.

Regarding the benefits related to feeding, the unidirectional behaviour of *P. tsukisome* sp. nov. and the position on the shell surfaces on the dorsal side of the host hermit crab while facing upward, might be adapted to facilitate consumption of the host’s faeces spilling from the shell aperture and the suspended particulate organic matter accumulating from the above water column. However, whether the actinopharyngeal position (Angle 1) is directly related to the feeding behaviour must be discussed as the position of the column (Angle 2) might benefit their feeding. The hypothesis on the feeding-related attaching positions has been previously proposed from the behavioural observation of other CF-sea anemones: *Stylobates calcifer*, which is also supposed to feed on the suspended particulate organic matter from the above water column, could actively climb to the host snail shells to the upward-facing position [12].

To understand the adaptive and evolutionary significances of its unidirectional behaviour, comparing closely related species focusing on CF ability and feeding ecology is necessary in the future.

### 4.8. Benefits of carcinoecium-forming association for the host hermit crab

The present study demonstrates that *O. monstrosus* has a significantly larger body size than some other non-species-specific symbiotic *Oncopagurus* species: specifically, *O. monstrosus* females were larger than 11 of the 20 species, and males were larger than 14 of the 24 species (figure 12A,B, electronic supplementary material, figure S4A,B, and table S8). Specimens of *O. monstrosus* definitively associated with *P. tsukisome* sp. nov. exhibited a significantly larger body size compared with those reported by Lemaitre [49], with an unidentified actiniarian species (figure 12C,D, electronic supplementary material, figure S4C,D). Considering that the growth and reproductive success of hermit crabs are influenced by shell availability [29–31], the larger body size of *O. monstrosus* than that of other species suggests that the carcinoecium probably functions as a portable shelter and/or prevents the degradation of snail shells. In particular, the CF ability of *P. tsukisome* sp. nov. may play a role in increasing the maximum growth size of its host hermit crab, either through a species-specific or an obligate symbiosis interaction. Furthermore, considering the larger body size of *O. monstrosus* than that of the sympatrically recorded species, *O. indicus* has an opportunistic association with Anthozoan polyps, strongly suggesting that *O. monstrosus* could achieve a larger body size in similar environments because of the carcinoecium produced by the new CF sea anemone. Thus, the findings of this study provide quantitative evidence supporting the adaptive benefits of the CF association for host hermit crabs.

Some hermit crab species, such as *O. africanus*, *O. gracilis*, *O. haigae* and *O. stockmani*, which are opportunistically symbiotic with Anthozoan polyps or Hydractinia colonies, do not exhibit significant size differences from *O. monstrosus* in either females or males. Although the Anthozoan species associated with these hermit crabs have not yet been identified, it is plausible that they are involved in symbiotic relationships with yet-undiscovered CF-associated Anthozoan species. Previous studies have reported that most CF associations are highly species-specific [7,10,13,16]. However, certain CF-associated sea anemones, such as *P. rosea*, *P. stephensoni* and *P. valdiviae*, have been found to associate with multiple hermit crab species, raising the possibility that these hermit crab species, *O. africanus*, *O. gracilis*, *O. haigae* and *O. stockman*, may also engage in unidentified CF associations [9,53,60,68].

Lemaitre [49] described *O. tuamotu* as ‘Gastropod shells, usually with Anthozoan polyps growing on the shell’ and so categorized this species to exhibit a strong association (e.g. species-specific or obligate symbiosis) with Anthozoan polyps included in the size comparison analysis, as well as *O. monstrosus*. However, Lemaitre examined only six specimens (three non-ovigerous females, one ovigerous female and two males) [49]. Therefore, whether this species has a species-specific association with Anthozoan species remains to be confirmed through continuous sampling in further study.

The observed trend in body size is more pronounced in males than in females (figure 12, electronic supplementary material, figure S4 and table S8) and may be attributed to the potential advantage that larger males have in male–male competition for mates’ choice and/or guard [95,96]. Consequently, larger males are likely to achieve greater reproductive success than the smaller individuals, reinforcing selection for increased body size in males.

The current findings, which demonstrate mutual benefits for both *P. tsukisome* sp. nov., and *O. monstrosus*, support a novel hypothesis regarding the evolution of CF ability and species-specific symbiosis in expansive deep-sea habitats. The CF ability of the symbiotic sea anemone may have evolved through a trade-off interaction, balancing investments in its own growth and reproductive success against the development of a chitinous coating on the snail shell inhabited by the host hermit crab, supported by the food supplied by the host. Positive selection may have impacted sea anemone individuals with well-developed chitinous coatings, driven by hermit crab symbiotic behaviours (e.g. shell-change frequency and the transfer of symbiotic sea anemones during shell changes). Meanwhile, these well-developed chitinous coatings may have allowed the hermit crabs to grow larger than closely related non-symbiotic species, thereby facilitating speciation in the deep-sea environment; perhaps, in addition to the above benefits, the lower shell-change frequency may also be energy-saving in the food-limited deep-sea environment and/or make them less vulnerable to opportunistic predators while changing shells. This trade-off relationship may have become more refined over time, potentially driving the evolution of species-specific symbiotic relationships.

This hypothesis could be tested by examining the relationship between *P. valdiviae* and *O. bicristatus*, which was described as being a non-species-specific but opportunistic association by Doumenc [53] and Lemaitre [49]. Focusing on the trade-off interactions and balance between symbiotic and non-symbiotic individuals in both species may provide further insights into the evolutionary dynamics of CF ability in sea anemones and species-specific relationships in both animal species on the deep-sea floor.

## 5. Conclusion

Herein, *Paracalliactis tsukisome* sp. nov. (Cnidaria: Actiniaria: Hormathiidae), a new sea anemone found on the Japanese deep-sea floor, is described. *Paracalliactis* species produce carcinoecium with a unidirectional trend that covers snail shells inhabited by the host hermit crab, *O. monstrosus*. This species differs in tentacle coloration, number of mesentery cycles, tubercle distribution, number of tentacles, and cnidae distribution in the column and mesenterial filament. Its phylogenetic position is supported by the data obtained for its mitochondrial genes 12S, 16S and COIII, and nuclear genes 18S and 28S.

The carbon (*δ*^13^C) and nitrogen (*δ*^15^N) stable isotopic analyses indicated that *P. tsukisome* sp. nov. may consume the faeces of the host as well as suspended particles from the surrounding environment. Moreover, three-dimensional CT analysis of this species revealed that a unidirectional attaching trend has evolved, as the siphonoglyph is always located near the shell aperture or carcinoecium edge, probably related to the feeding behaviour or CF formation of this new species. Furthermore, the findings demonstrate that host hermit crabs benefit from the association, as *O. monstrosus* has a significantly bigger body size than other *Oncopagurus* species. The carcinoecium produced by *P. tsukisome* sp. nov. may effectively function as the host’s shell on the deep-sea floor. These results provide the first quantitative evidence of mutualism in CF associations, thus outlining a remarkable example of symbiotic relationships in the deep sea. These findings offer valuable insights into interspecific interactions that drive the evolution of unique traits within deep-sea-floor communities.

## 6. Identification key for *Paracalliactis* species

**a1) Scapus and scapulus well separated, unclearly divided, strongly introverted, or with a short scapulus and/or with tubercles → b**

**b1**) One size range of basitrichs in tentacles → **c**
  **c1**) One size range of basitrichs in acontia → **d**
    **d1**) Scapus and scapulus are unclearly divided, incomplete corona of tubercles, one size range of spirocyst in tentacles, one size range of basitrich in Actinopharynx; distributed on the Pacific side of central Honshu Island, Japan (the northwestern Pacific Ocean) (192–470 m).
    **→*****Paracalliactis tsukisome*** **sp. nov.**
    **d2**) Scapus and scapulus are well divided, complete corona of tubercles, two size range of spirocyst in tentacles, two size range of basitrich in Actinopharynx; distributed in the North Atlantic Ocean (4166–4700 m).
    **→*****Paracalliactis valdiviae*** **Carlgren, 1928**
  **c2**) Two size range of basitrichs in acontia → **e**
    **e1**) Two size range of spirocyst in tentacles and distributed in the North Atlantic Ocean depth range 375−550 m
    → ***Paracalliactis obvolva*** **Carlgren, 1928**
    **e2**) One size range of spirocyst in tentacles → **f**
      **f1**) Tentacle with *b*-mastigophore and distributed in the western Atlantic Ocean and the eastern Pacific Ocean depth range 600−660 m
      →***Paracalliactis consors*** **(Verrill, 1882)**
      **f2**) Tentacle without *b*-mastigophore and distributed in the North Atlantic Ocean depth range 1340−2700 m
      →***Paracalliactis stephensoni*** **Carlgren, 1928**
**b2**) Two size range of basitrichs in tentacles → **g**
  **g1**) Two size range of basitrichs in acontia
  →*Paracalliactis rosea* **Hand, 1976**
  **g2**) One size range of basitrichs in acontia → **h**
    **h1**) Distributed in the North Atlantic Ocean depth range 2900−3800 m
    →***Paracalliactis azorica*** **Doumenc, 1975**
    **h2**) Distributed in the Northwestern Pacific Ocean depth range 39−47 m
    →***Paracalliactis sinica*** **Pei, 1982**

**a2**) **Scapus and scapulus well separated, without tubercles → i**

**i1)** More than 100 tentacles (up to 172)
→***Paracalliactis*** **sp. (ms. n., described as** ***P. niwa*** **by Gusmão [9])**
**i2**) Tentacles 90−96, two size range of spirocyst in tentacles, oral disc facing upward (dorsal side of host hermit crab); distributed along the coast of Somaliland, Indian Ocean (628–823 m).
**→*****Paracalliactis michaelsarsi*** **Carlgren, 1928**

## Data Availability

The videos depicting the expansion–contraction behaviour of *Paracalliactis tsukisome* sp. nov. is appended as appendix videos. Genetic data can be obtained from Genbank (accession nos. LC643556-LC643567: https://www.ncbi.nlm.nih.gov/genbank). Supplementary material is available online [97].

## References

[1] Darwin C. 1859 On the origin of species. London, UK: Murray.

[2] Izumi T, Ise Y, Yanagi K, Shibata D, Ueshima R. 2018 First detailed record of symbiosis between a sea anemone and homoscleromorph sponge, with a description of Tempuractis rinkai gen. et sp. nov. (Cnidaria: Anthozoa: Actiniaria: Edwardsiidae). Zool. Sci. **35**, 188–198. (10.2108/zs170042) 29623791

[3] Wiebes JT. 1979 Co-evolution of figs and their insect pollinators. Annu. Rev. Ecol. Syst. **10**, 1–12. (10.1146/annurev.es.10.110179.000245)

[4] Darwin C. 1862 On the various contrivances by which British and foreign orchids are fertilized by insects, and on the good effects of intercrossing. London, UK: Murray. PMC5180317 30163543

[5] Nilsson LA. 1988 The evolution of flowers with deep corolla tubes. Nature **334**, 147–149. (10.1038/334147a0)

[6] Thompson JN. 1994 The co-evolutionary process. Chicago, IL, USA: University of Chicago Press.

[7] Crowther AL, Fautin DG, Wallace CC. 2011 Stylobates birtlesi sp. n., a new species of carcinoecium-forming sea anemone (Cnidaria, Actiniaria, Actiniidae) from eastern Australia. Zookeys **89**, 33–48. (10.3897/zookeys.89.825) PMC3082962 21594082

[8] Daly M, Ardelean A, Cha HR, Campbell AC, Fautin DG. 2004 A new species, Adamsia obvolva (Cnidaria: Anthozoa: Actiniaria), from the Gulf of Mexico, and a discussion of the taxonomy of carcinoecium-forming sea anemones. Bull. Mar. Sci. **74**, 385–399.

[9] Gusmão LC. 2010 Systematics and evolution of sea anemones (Cnidaria: Actiniaria: Hormathiidae) symbiotic with hermit crabs. Electronic Thesis or Dissertation, Ohio State University, Columbus, OH, USA. 10.1016/j.ympev.2010.05.001 20457262

[10] Gusmão LC, Van Deusen V, Daly M, Rodríguez E. 2020 Origin and evolution of the symbiosis between sea anemones (Cnidaria, Anthozoa, Actiniaria) and hermit crabs, with additional notes on anemone-gastropod associations. Mol. Phylogenet. Evol. **148**, 106805. (10.1016/j.ympev.2020.106805) 32217169

[11] Ranjith L *et al*. 2025 Symbiosis in sea anemones: a few case reports along the Bay of Bengal Large Marine Ecosystem. Mar. Ecol. **46**, e12855. (10.1111/maec.12855)

[12] Antoniadou C, Vafeiadou AM, Chintiroglou C. 2013 Symbiosis of sea anemones and hermit crabs in temperate seas. In Symbiosis, evolution, biology and ecological effects (eds AF Camisão, CC Pedroso), pp. 95–117. New York, NY, USA: NOVA Science.

[13] Yoshikawa A, Izumi T, Moritaki T, Kimura T, Yanagi K. 2022 Carcinoecium-forming sea anemone Stylobates calcifer sp. nov. (Cnidaria, Actiniaria, Actiniidae) from the Japanese deep-sea floor: a taxonomical description with its ecological observations. Biol. Bull. **242**, 127–152. (10.1086/719160) 35580031

[14] Dunn DF, Liberman MH. 1983 Chitin in sea anemone shells. Science **221**, 157–159. (10.1126/science.221.4606.157) 17769214

[15] Gusmão LC, Daly M. 2010 Evolution of sea anemones (Cnidaria: Actiniaria: Hormathiidae) symbiotic with hermit crabs. Mol. Phylogenet. Evol. **56**, 868–877. (10.1016/j.ympev.2010.05.001) 20457262

[16] Yoshikawa A, Nakazawa S, Asakura A. 2019 A brief description of surface structure and composition of the pseudo-snail shell formed by a sea anemone Stylobates sp. symbiotic with hermit crabs from the deep-sea floor. Zool. Sci. **36**, 284–293. (10.2108/zs180167) 34664898

[17] Noshita K, Shimizu K, Sasaki T. 2016 Geometric analysis and estimation of the growth rate gradient on gastropod shells. J. Theor. Biol. **389**, 11–19. (10.1016/j.jtbi.2015.10.011) 26506470

[18] Genikhovich G, Technau U. 2017 On the evolution of bilaterality. Development **144**, 3392–3404. (10.1242/dev.141507) 28974637

[19] Carlgren O. 1949 A survey of the Ptychodactiaria, Corallimorpharia and Actiniaria, Kungl. Sven. Vetenskapsakademiens Handl. **1**, 1–121.

[20] Malakhov VV. 2016 Symmetry and the tentacular apparatus in Cnidaria. Russ. J. Mar. Biol. **42**, 287–298. (10.1134/s1063074016040064)

[21] Przeslawski R, Christenhusz MJM. 2022 Deep-sea discoveries. Zool. J. Linn. Soc. **194**, 1037–1043. (10.1093/zoolinnean/zlac022)

[22] Dunn DF, Devaney DM, Roth R. 1980 Stylobates: a shell-forming sea anemone (Coelenterata, Anthozoa, Actiniidae). Pac. Sci. **34**, 379–388.

[23] MacIntosh H *et al*. 2018 Invertebrate diversity in the deep Great Australian Bight (200–5000 m). Mar. Biodivers. Rec. **11**, 23. (10.1186/s41200-018-0158-x)

[24] O’Hara TD *et al*. 2020 The lower bathyal and abyssal seafloor fauna of eastern Australia. Mar. Biodivers. Rec. **13**, 11. (10.1186/s41200-020-00194-1)

[25] Stachowitsch M. 1979 Movement, activity pattern, and rôle of a hermit crab population in a sublittoral epifaunal community. J. Exp. Mar. Biol. Ecol. **39**, 135–150. (10.1016/0022-0981(79)90010-8)

[26] Stachowitsch M. 1980 The epibiotic and endolithic species associated with the gastropod shells inhabited by the hermit crabs Paguristes Oculatus and Pagurus Cuanensis. Mar. Ecol. **1**, 73–101. (10.1111/j.1439-0485.1980.tb00223.x)

[27] Chintiroglou C, Koukouras A. 1991 Observations on the feeding habits of Calliactis parasitica (Couch, 1842) (Anthozoa, Cnidaria). Ocean. Acta **14**, 389–396.

[28] Reese ES. 1969 Behavioral adaptations of intertidal hermit crabs. Am. Zool. **9**, 343–355. (10.1093/icb/9.2.343)

[29] Wilber TP. 1989 Associations between gastropod shell characteristics and egg production in the hermit crab Pagurus longicarpus. Oecologia **81**, 6–15. (10.1007/BF00377002) 28312149

[30] Asakura A. 1991 Population ecology of the sand-dwelling hermit crab Diogenes nitidimanus. IV Larval settlement. Mar. Ecol. Prog. Ser. **78**, 139–146. (10.3354/meps078139)

[31] Presnell JK, Schreibman MP. 1997 Humason’s animal tissue techniques, 5th edn. Baltimore, MD, USA: Johns Hopkins University Press.

[32] Yanagi K, Fujii T, Hirose M. 2015 Redescription of the sea anemone Exocoelactis actinostoloides (Cnidaria: Anthozoa: Actiniaria) based on a topotypic specimen collected from Tokyo Bay, Japan. Spec. Div. **20**, 199–209. (10.12782/sd.20.2.199)

[33] Mariscal RN. 1974 Nematocysts. In Coelenterate biology: reviews and new perspectives (eds L Muscatine, HM Lenhoff), pp. 129–178. New York, NY, USA: Academic Press. (10.1016/B978-0-12-512150-7.50008-6)

[34] Abramoff MD, Magelhães PJ, Ram SJ. 2004 Image processing with ImageJ. Biophotonics Int. **11**, 36–42.

[35] Katoh K, Standley DM. 2013 MAFFT multiple sequence alignment software version 7: improvements in performance and usability. Mol. Biol. Evol. **30**, 772–780. (10.1093/molbev/mst010) 23329690 PMC3603318

[36] Castresana J. 2002 Gblocks server. See http://molevol.cmima.csic.es/castresana/Gblocks_server.html.

[37] Tanabe AS. 2011 Kakusan4 and Aminosan: two programs for comparing nonpartitioned, proportional and separate models for combined molecular phylogenetic analyses of multilocus sequence data. Mol. Ecol. Resour. **11**, 914–921. (10.1111/j.1755-0998.2011.03021.x) 21592310

[38] Stamatakis A. 2006 RAxML-VI-HPC: maximum likelihood-based phylogenetic analyses with thousands of taxa and mixed models. Bioinformatics **22**, 2688–2690. (10.1093/bioinformatics/btl446) 16928733

[39] Ronquist F, Huelsenbeck JP. 2003 MrBayes 3: Bayesian phylogenetic inference under mixed models. Bioinformatics **19**, 1572–1574. (10.1093/bioinformatics/btg180) 12912839

[40] Rambaut A. 2018 Figtree ver 1.4.4*.* Edinburgh, UK: Institute of Evolutionary Biology, University of Edinburgh.

[41] Kimura M. 1980 A simple method for estimating evolutionary rates of base substitutions through comparative studies of nucleotide sequences. J. Mol. Evol. **16**, 111–120. (10.1007/BF01731581) 7463489

[42] Kumar S, Stecher G, Li M, Knyaz C, Tamura K. 2018 MEGA X: molecular evolutionary genetics analysis across computing platforms. Mol. Biol. Evol. **35**, 1547–1549. (10.1093/molbev/msy096) 29722887 PMC5967553

[43] Bligh EG, Dyer WJ. 1959 A rapid method of total lipid extraction and purification. Can. J. Biochem. Physiol. **37**, 911–917. (10.1139/o59-099) 13671378

[44] Zhao L *et al*. 2023 Ocean acidification stunts molluscan growth at CO_2_ seeps. Sci. Total Environ. **873**, 162293. (10.1016/j.scitotenv.2023.162293) 36813205

[45] Nishimoto A, Mito S, Shirayama Y. 2009 Organic carbon and nitrogen source of sunken wood communities on continental shelves around Japan inferred from stable isotope ratios. Deep Sea Res. II **56**, 1683–1688. (10.1016/j.dsr2.2009.05.032)

[46] Reid REB, Crowley BE, Haupt RJ. 2023 The prospects of poop: a review of past achievements and future possibilities in faecal isotope analysis. Biol. Rev. Camb. Philos. Soc. **98**, 2091–2113. (10.1111/brv.12996) 37438959

[47] Cignoni P, Callieri M, Corsini M, Dellepiane M, Ganovelli F, Ranzuglia G. 2008 MeshLab: an open-source mesh processing tool. In Italian Chapter Conf, pp. 129–136. (10.2312/LocalChapterEvents/ItalChap/ItalianChapConf2008/129-136)

[48] Bezanson J, Edelman A, Karpinski S, Shah VB. 2017 Julia: a fresh approach to numerical computing. SIAM Rev. **59**, 65–98. (10.1137/141000671)

[49] Lemaitre R. 2014 A worldwide taxonomic and distributional synthesis of the genus Oncopagurus Lemaitre, 1966 (Crustacea: Decapoda: Anomura: Parapaguridae), with descriptions of nine new species. Raffles Bull. Zool. **62**, 210–301.

[50] Lemaitre R. 1996 Hermit crabs of the family Parapaguridae (Crustacea: Decapoda: Anomura) from Australia: species of Strobopagurus Lemaitre, 1989, Sympagurus Smith, 1883, and two new genera. Rec. Aust. Mus. **48**, 163–221. (10.3853/j.0067-1975.48.1996.286)

[51] Lemaitre R, McLaughlin PA. 1992 Descriptions of megalopa and juveniles of Sympagurus dimorphus (Studer, 1883), with an account of the Parapaguridae (Crustacea: Anomura: Paguroidea) from Antarctic and Subantarctic waters. J. Nat. Hist. **26**, 745–768. (10.1080/00222939200770471)

[52] Zhadan DG. 1997 Deep-sea hermit crabs from the submerged ridges Nazka and Sala-y-Gomez, southeastern Pacific (Decapoda Anomura Parapaguridae). Arthropoda Sel. **6**, 55–79.

[53] Doumenc D. 1975 Actinies bathyales et abyssales de l’océan Atlantique nord Familles des Hormathiidae (genres Paracalliactis et Phelliactis) et des Actinostolidae (genres Actinoscyphia et Sicyonis). Bulletin Du Muséum National d’histoire Naturelle **287**, 157–204. (10.5962/p.279151)

[54] R Core Team. 2023 *R: a language and environment for statistical computing*. R Foundation for Statistical Computing. See https:// www.R-project.org/.

[55] Gusmão LC, Rodríguez E, Daly M. 2019 Description of Calliactis tigris sp. nov.: reconciling taxonomy and phylogeny in hermit-crab symbiotic anemones (Cnidaria: Actiniaria: Hormathiidae). Org. Divers. Evol. **19**, 567–583. (10.1007/s13127-019-00414-2)

[56] Sanamyan NP, Sanamyan KE, Bocharova ES, Morozov TB, Galkin SV. 2023 Sea anemones (Actiniaria, Corallimorpharia and Zoantharia) from the Western Bering Sea (Northwest Pacific). Invert. Zool. **20**, 27–56. (10.15298/invertzool.20.1.02)

[57] McMurrich JP. 1893 Report on the Actiniæ collected by the United States Fish Commission steamer Albatross during the winter of 1887–1888. Proc. U.S. Natl. Mus. **16**, 119–216.

[58] Carlgren O. 1947 Further contributions to a revision of the Actiniaria and Corallimorpharia. Ark. Zool. **17**, 1–17.

[59] Carlgren O. 1928 Zur Symbiose zwischen Actinien und Paguriden. Z. Morph. u. Okol. Tiere. **12**, 165–173. (10.1007/BF00407632)

[60] Hand C. 1975 Behaviour of some New Zealand sea anemones and their molluscan and crustacean hosts. N. Z. J. Mar. Freshw. Res. **9**, 509–527. (10.1080/00288330.1975.9515585)

[61] Pei Z. 1982 A new species of the genus Paracalliactis (Hormathiidae: Actiniaria) from the East China Sea. Stud. Mar. Sin. **19**, 69–71.

[62] Carlgren O. 1945 Further contributions to the knowledge of the cnidom in the Anthozoa especially in the Actiniaria. K. Fysiogr. Sällsk. Handl. **56**, 1–24.

[63] Carlgren O. 1928 Actiniaria der Deutschen Tiefsee-Expedition. Wiss. Ergeb. Dtsch. Tiefsee-Exped. **22**, 123–266.

[64] Izumi T, Fujii T. 2021 Gems of the southern Japanese seas – four new species of Edwardsianthus (Anthozoa, Actiniaria, Edwardsiidae) with redescriptions of two species. Zookeys **1076**, 151–182. (10.3897/zookeys.1076.69025) 35002358 PMC8683394

[65] Schama R, Mitchell M, Solé-Cava AM. 2012 Actinia ebhayiensis sp. nov., a new species of sea anemone (Anthozoa: Actiniaria: Actiniidae) from South Africa. J. Mar. Biol. Assoc. U. K. **92**, 885–894. (10.1017/s0025315411001305)

[66] Stevens M, Merilaita S. 2009 Animal camouflage: current issues and new perspectives. Phil. Trans. R. Soc. B **364**, 423–427. (10.1098/rstb.2008.0217) 18990674 PMC2674078

[67] Detto T, Backwell PRY, Hemmi JM, Zeil J. 2006 Visually mediated species and neighbour recognition in fiddler crabs (Uca mjoebergi and Uca capricornis). Proc. R. Soc. B **273**, 1661–1666. (10.1098/rspb.2006.3503) PMC1634930 16769638

[68] Detto T, Hemmi JM, Backwell PRY. 2008 Colouration and colour changes of the fiddler crab, uca capricornis: a descriptive study. PLoS One **3**, e1629. (10.1371/journal.pone.0001629) 18286186 PMC2229841

[69] Randall JE. 2005 A review of mimicry in marine fishes. Zool. Stud. **44**, 299–328.

[70] Silbiger N, Munguia P. 2008 Carapace color change in Uca pugilator as a response to temperature. J. Exp. Mar. Biol. Ecol. **355**, 41–46. (10.1016/j.jembe.2007.11.014)

[71] Kronstadt SM, Darnell MZ, Munguia P. 2013 Background and temperature effects on Uca panacea color change. Mar. Biol. **160**, 1373–1381. (10.1007/s00227-013-2189-5)

[72] Russell BJ, Dierssen HM. 2018 Color change in the Sargassum crab, Portunus sayi: response to diel illumination cycle and background albedo. Mar. Biol. **165**, 28. (10.1007/s00227-018-3287-1)

[73] Green SD, Duarte RC, Kellett E, Alagaratnam N, Stevens M. 2019 Colour change and behavioural choice facilitate chameleon prawn camouflage against different seaweed backgrounds. Commun. Biol. **2**, 230. (10.1038/s42003-019-0465-8) 31263774 PMC6588621

[74] Yoshikawa A, Yasuda A, Izumi T, Yanagi K. 2022 A novel epibiotic association in the benthic community: the sea anemone Verrillactis sp. (Actiniaria: Sagartiidae) on the necto-benthic fish, Inimicus japonicus. Plankton Benthos Res. **17**, 208–213. (10.3800/pbr.17.208)

[75] Gusmão LC, Grajales A, Rodríguez E. 2018 Sea anemones through X-rays: visualization of two species of Diadumene (Cnidaria, Actiniaria) using micro-CT. Am. Museum Nov. **3907**, 1–47. (10.1206/3907.1)

[76] Cutress CE. 1955 An interpretation of the structure and distribution of cnidae in Anthozoa. Syst. Zool. **4**, 120. (10.2307/2411864)

[77] Östman C. 2000 A guideline to nematocyst nomenclature and classification, and some notes on the systematic value of nematocysts. Sci. Mar. **64**, 31–46. (10.3989/scimar.2000.64s131)

[78] Schmidt H. 1969 Die Nesselkapseln der Aktinien und ihre differentialdiagnostische Bedeutung. Helgolander Wiss. Meeresunters. **19**, 284–317. (10.1007/BF01625610)

[79] Weill R. 1934 Contribution à l’étude des cnidaires et de leurs nématocystes I, II Travaux de la Station. Zool de Wimereux **10–11**, 1–701.

[80] England KW. 1991 Nematocysts of sea anemones (Actiniaria, Ceriantharia and Corallimorpharia: Cnidaria): nomenclature. Hydrobiologia **216**, 691–697. (10.1007/BF00026532)

[81] Kimura T. 2019 Benthic deep-sea fauna in the Sea of Kumano, Mie Prefecture, Japan. Ann. Field Res. Technol. Mie Univ. **16**, 1–32.

[82] Asakura A, Watabe H, Hashimoto J. 2006 Recent topics on taxonomy of hermit crabs from Japanese waters – family Parapaguridae part II. Aquabiology **28**, 211–226.

[83] Baba K, Hayashi KI, Toriyama M. 1986 Decapod crustaceans from continental shelf and slope around japan. Tokyo, Japan: Japan Fisheries Resource Conservation Association, Tosho Printing Co., Ltd.

[84] Ikeda. 2013 Annual report of Okinawa Churaumi Aquarium No. 8, April 2010–March 2012.

[85] Miyake S. 1982 Japanese crustacean decapods and stomatopods in color. In Macrura, anomura, and stomatopoda, p. 261, vol. 1. Osaka, Japan: Hoikusha Publishing Co., Ltd.

[86] Henderson JR. 1896 Natural history notes from HM Indian marine survey steamer ‘investigator’, commander CF Oldham, RN, commanding, ser. 2, no. 24. Report on the Paguridae collected during the season 1893-94. J. Asiatic Soc. Bengal **65**, 516–536.

[87] Alcock A. 1901 A descriptive catalogue of the Indian deep-sea Crustacea Decapoda Macrura and Anomala in the Indian Museum, being a revised account of the decapod species collected by the Royal Indian marine survey ship investigator, p. 286. Calcutta, India: Printed by order of the trustees of the Indian Museum.

[88] Kemp SW, Sewell SRB. 1912 Notes on the Decapoda in the Indian Museum III. The species obtained by the R.I.M.S.S. 'investigator' during the survey season 1910-1911. Rec. Indian Mus. **7**, 15–32. (10.5962/bhl.part.28226)

[89] McLaughlin PA. 2002 A review of the hermit-crab (Decapoda: Anomura: Paguridea) fauna of southern Thailand, with particular emphasis on the Andaman Sea, and descriptions of three new species. Phuket Mar. Biol. Cent. Spec. Publ. **23**, 385–460.

[90] McLaughlin PA, Rahayu DL, Komai T, Chan TY. 2007 A catalog of the hermit crabs (Paguroidea) of Taiwan. Keelung, Taiwan: National Taiwan Ocean University.

[91] Post DM, Pace ML, Hairston NG. 2000 Ecosystem size determines food-chain length in lakes. Nature **405**, 1047–1049. (10.1038/35016565) 10890443

[92] Gorokhova E, Hansson S. 1999 An experimental study on variations in stable carbon and nitrogen isotope fractionation during growth of Mysis mixta and Neomysis integer. Can. J. Fish. Aquat. Sci. **56**, 2203–2210. (10.1139/f99-149)

[93] Dall WH. 1903 A new genus of Trochidae. Nautilus **17**, 61–62. (10.5962/bhl.part.18320)

[94] Fautin DG. 1987 Stylobates loisetteae, a new species of shell-forming sea anemone (Coelenterata: Actiniidae) from Western Australia. Proc. Calif. Acad. Sci. **45**, 1–7.

[95] Harvey AW. 1990 Sexual differences in contemporary selection acting on size in the hermit crab Clibanarius digueti. Am. Nat. **136**, 292–304. (10.1086/285099)

[96] Yoshino K, Koga T, Oki S. 2011 Chelipeds are the real weapon: cheliped size is a more effective determinant than body size in male–male competition for mates in a hermit crab. Behav. Ecol. Sociobiol. **65**, 1825–1832. (10.1007/s00265-011-1190-6)

[97] Yoshikawa A *et al*. 2025 Supplementary material from: Mutualism on the deep-sea floor: a novel shell-forming sea anemone in symbiosis with a hermit crab. Figshare. (10.6084/m9.figshare.c.8039810) PMC12539969 41127793

